# Supplementary material for: MiR-21 mediates the protection of kaempferol against hypoxia/reoxygenation-induced cardiomyocyte injury via promoting Notch1/PTEN/AKT signaling pathway
Source: PLoS One. 2020 Nov 5;15(11):e0241007. doi: 10.1371/journal.pone.0241007 (PMC7644004; doi:10.1371/journal.pone.0241007)
Supplement: S1 File — (DOC) [file pone.0241007.s001.doc]

Full-length blots/gels are presented in Supplementary Figure 1E

Each experiment was repeated three times


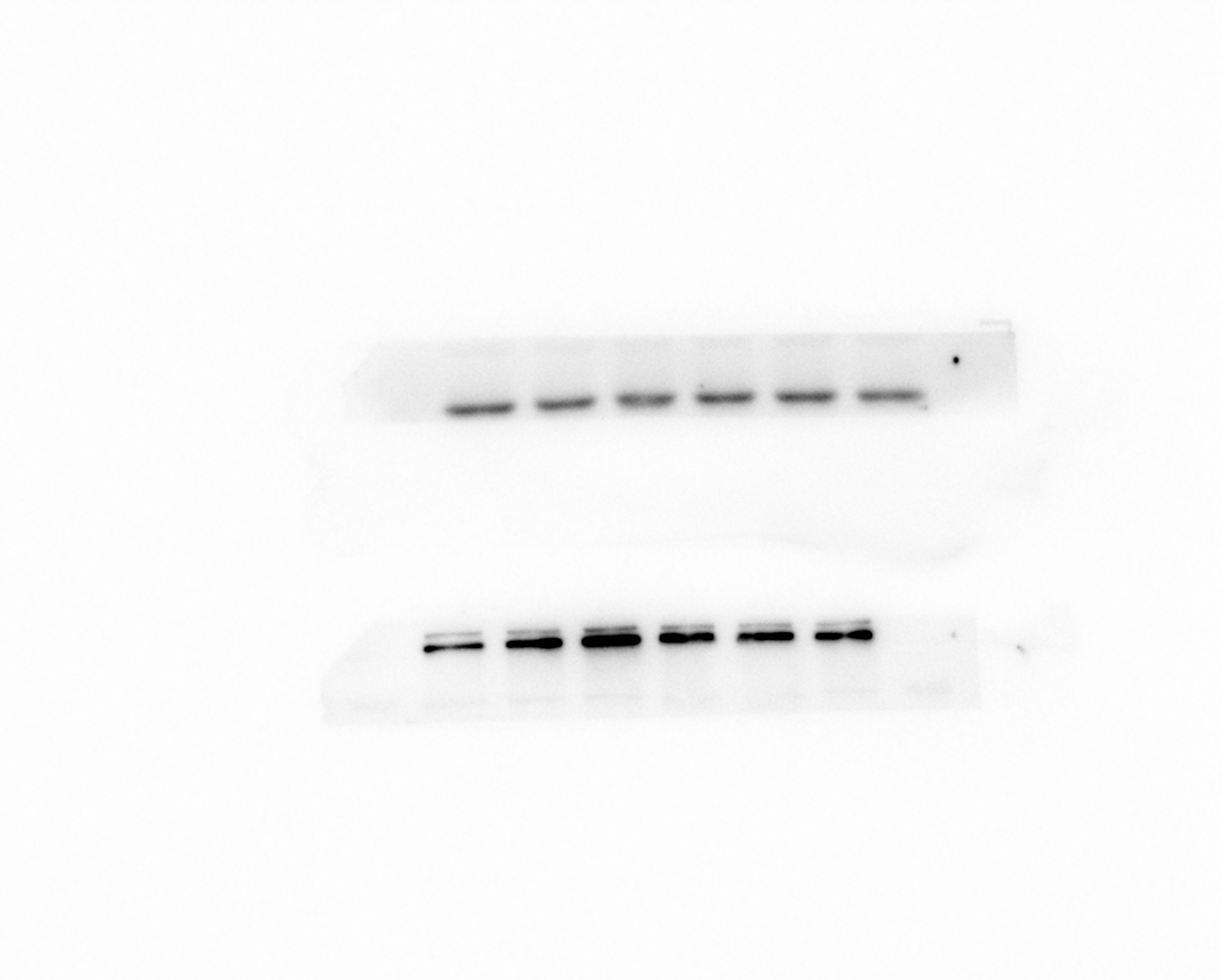


Bax (Lower band, left-to-right):

marker,

control,

H/R,

Kae + H/R,

Kae，

marker


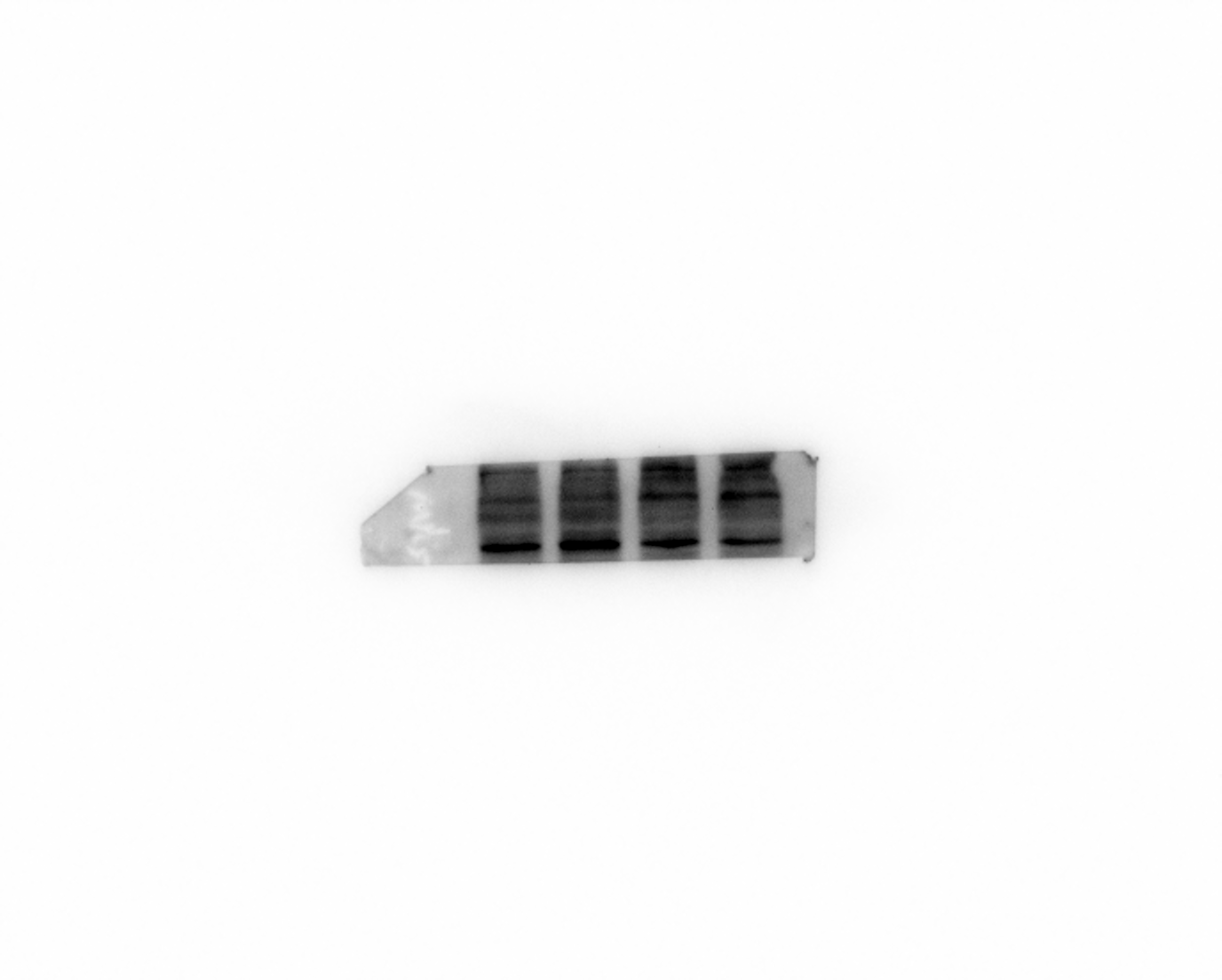


Bax (left-to-right):

control,

H/R,

Kae + H/R,

Kae，


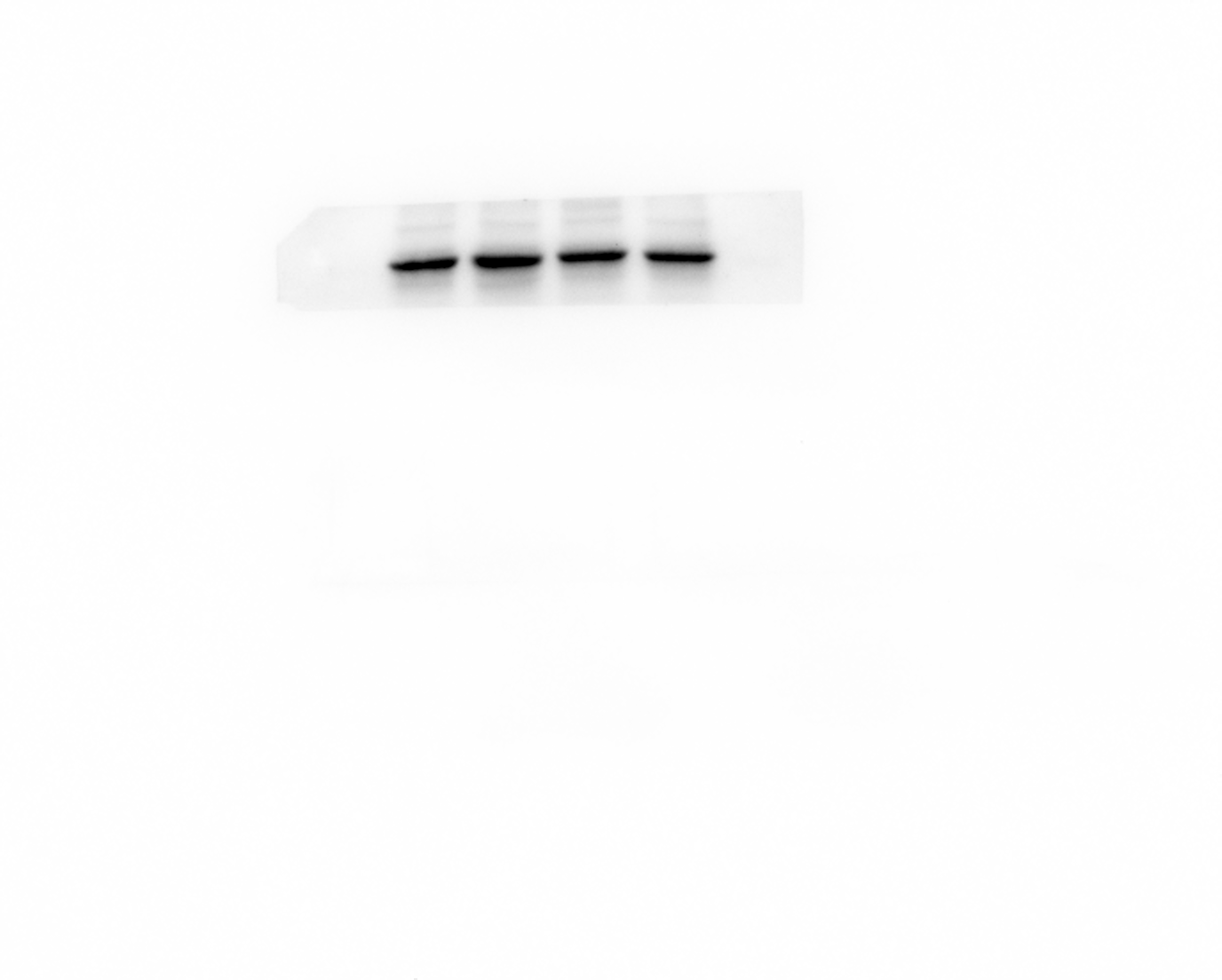


Bax (left-to-right):

control,

H/R,

Kae + H/R,

Kae
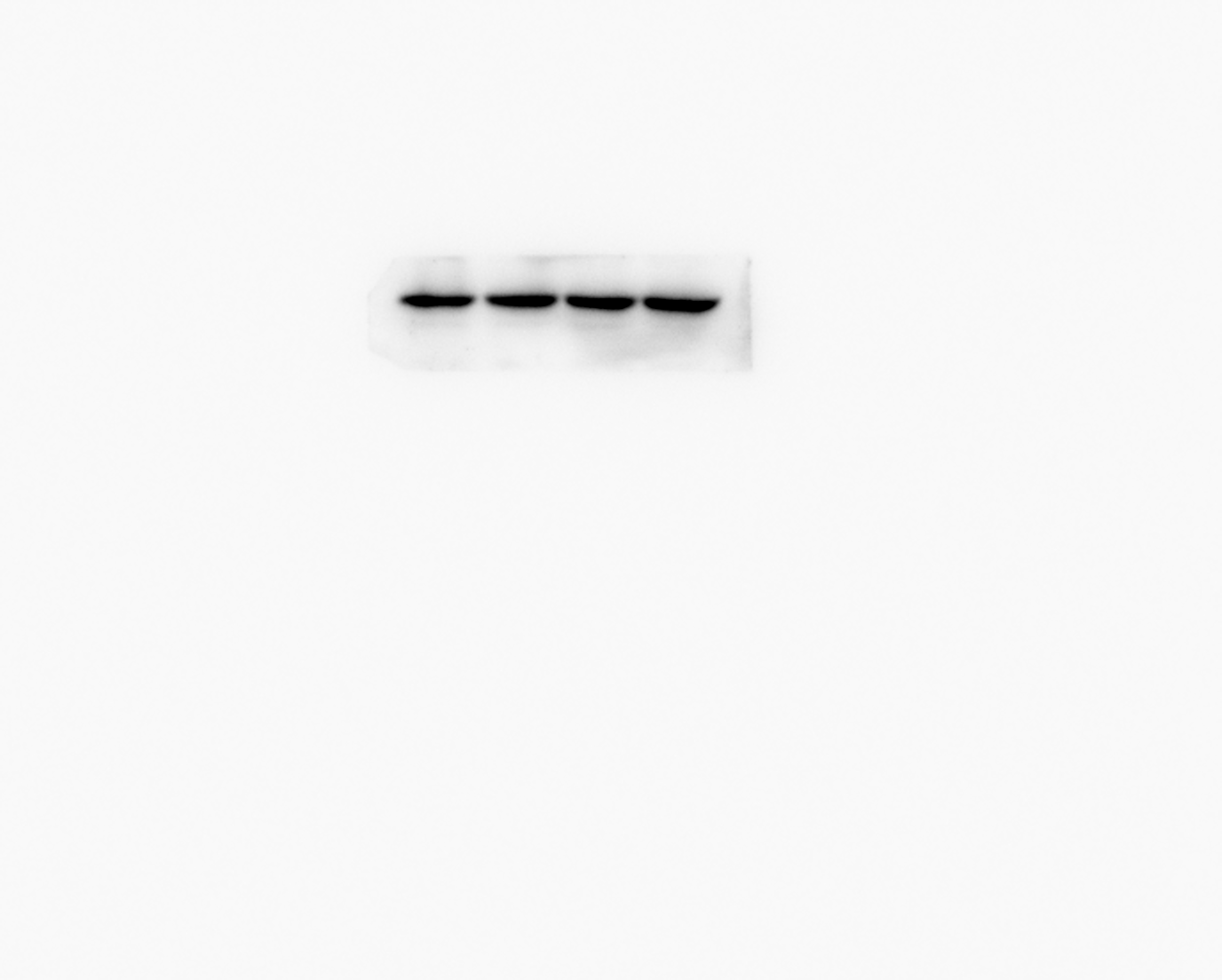
，


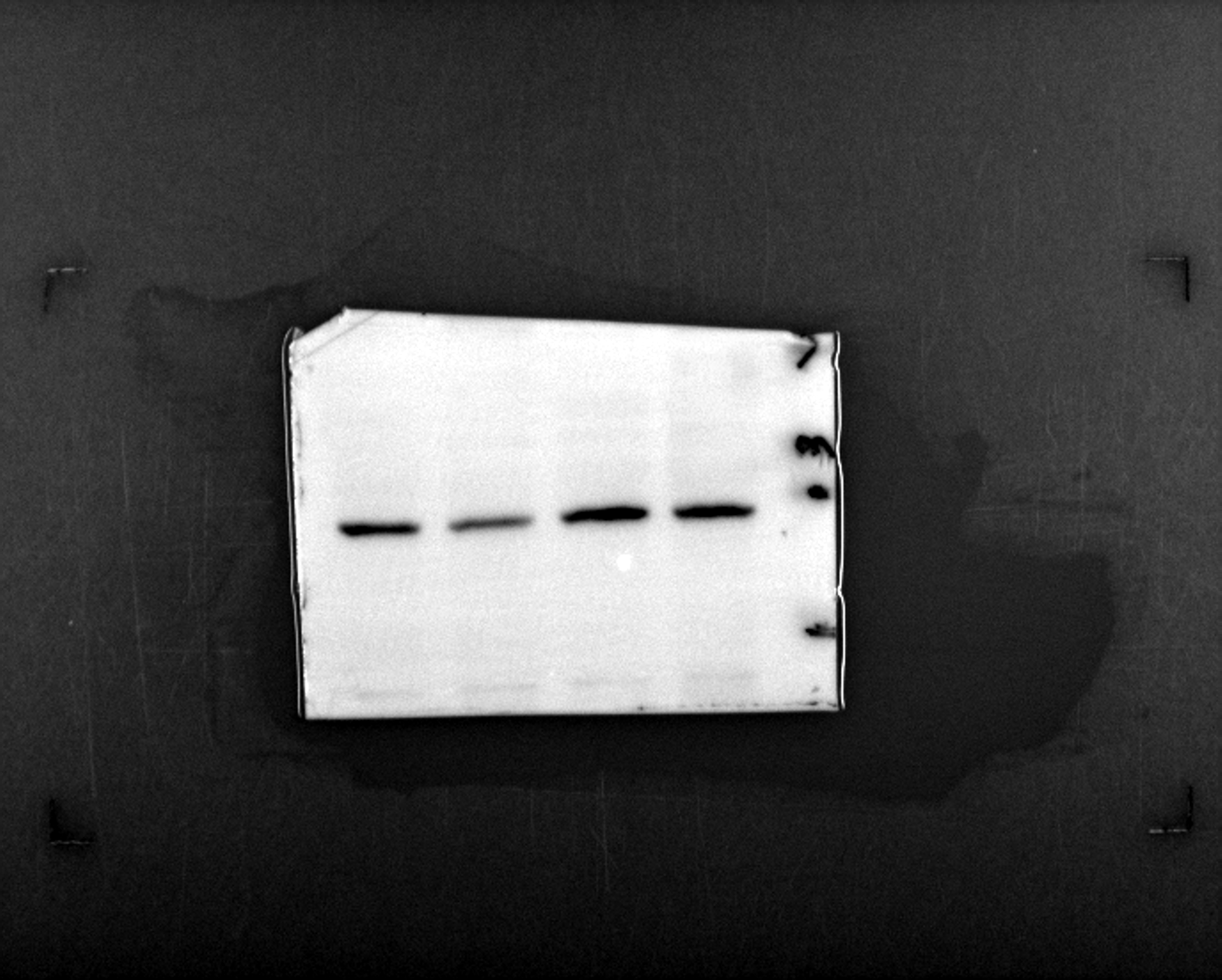


Bcl-2 (left-to-right):

control,

H/R,

Kae + H/R,

Kae，


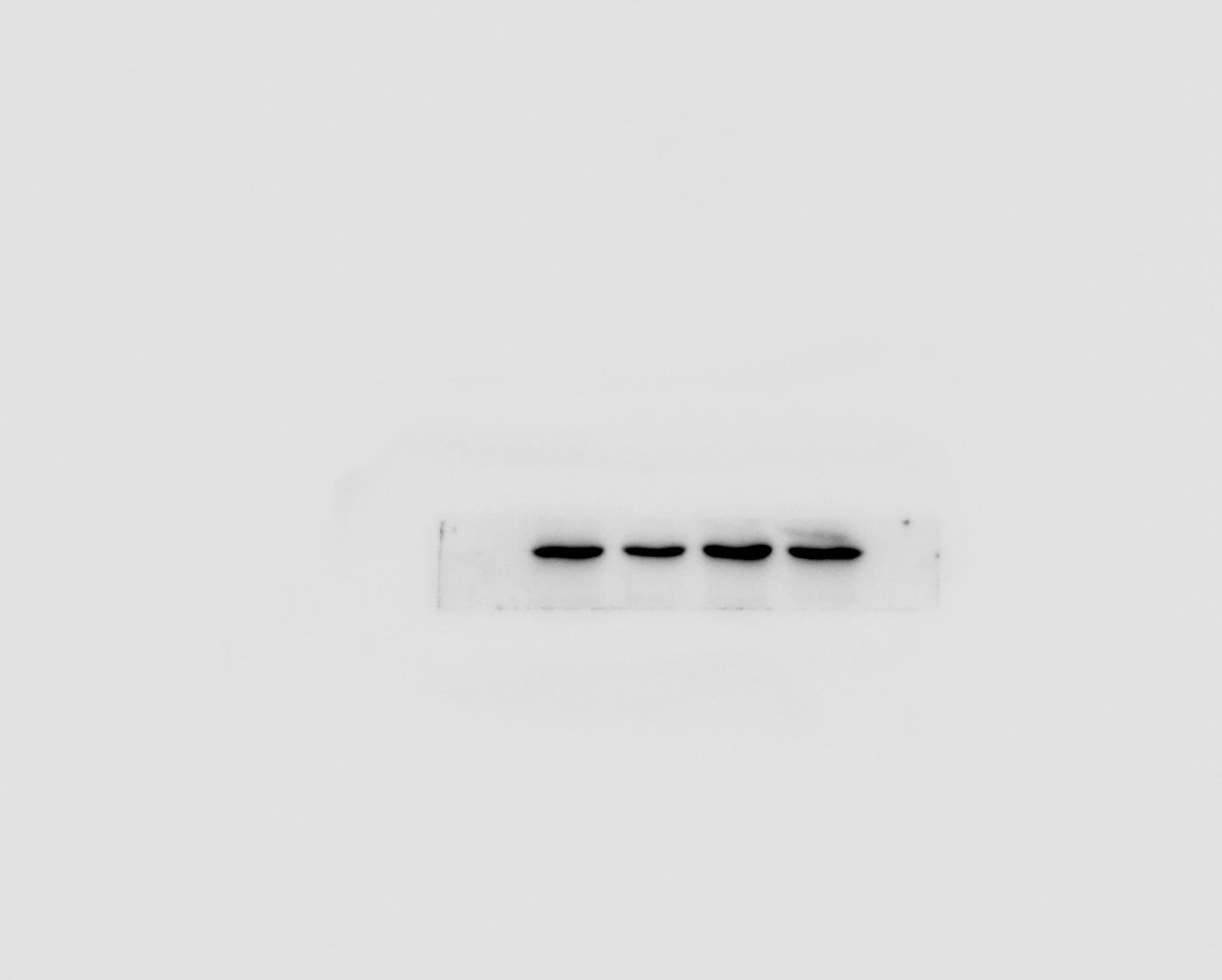


Bcl-2 (left-to-right):

control,

H/R,

Kae + H/R,

Kae，


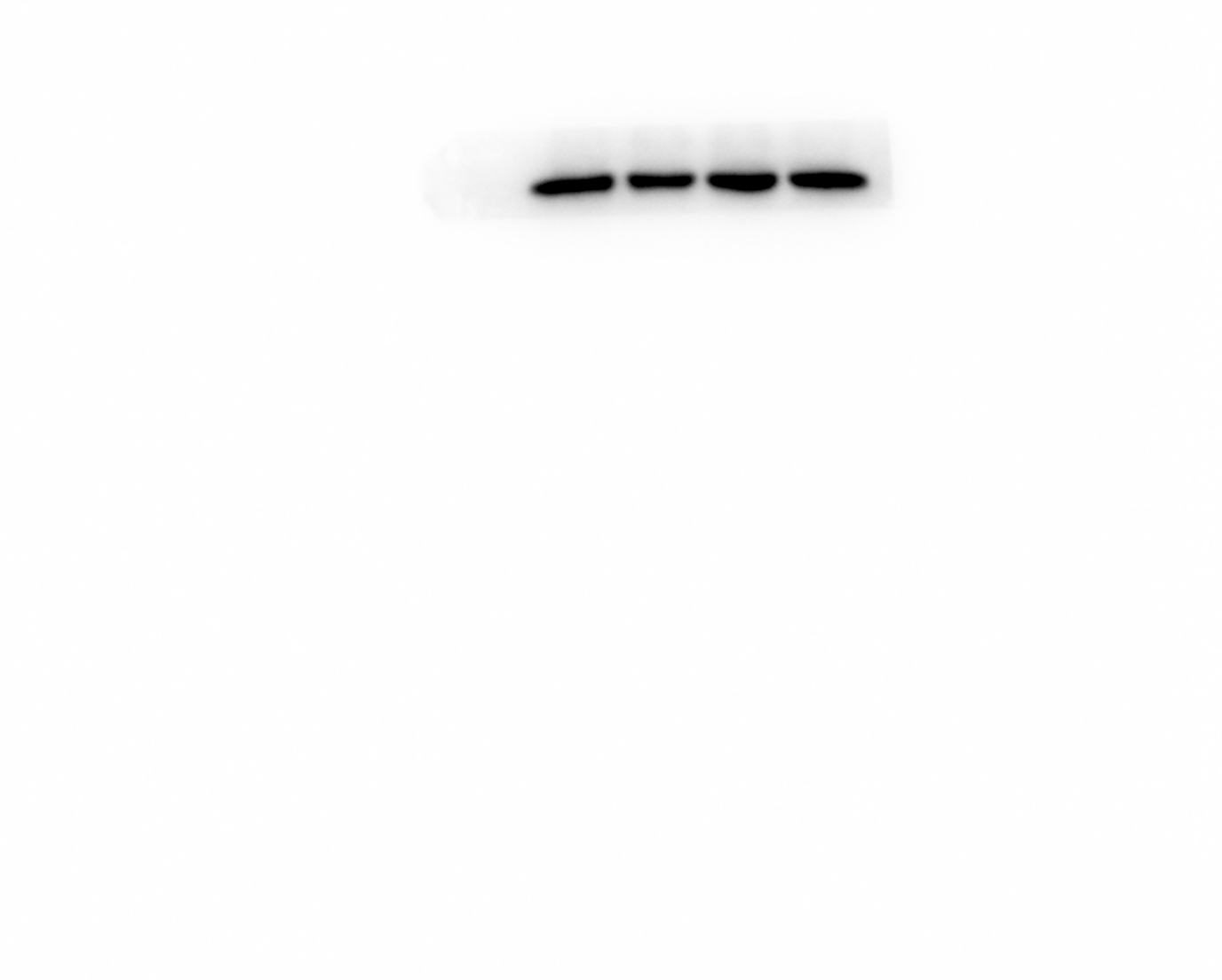


Bcl-2 (left-to-right):

control,

H/R,

Kae + H/R,

Kae，


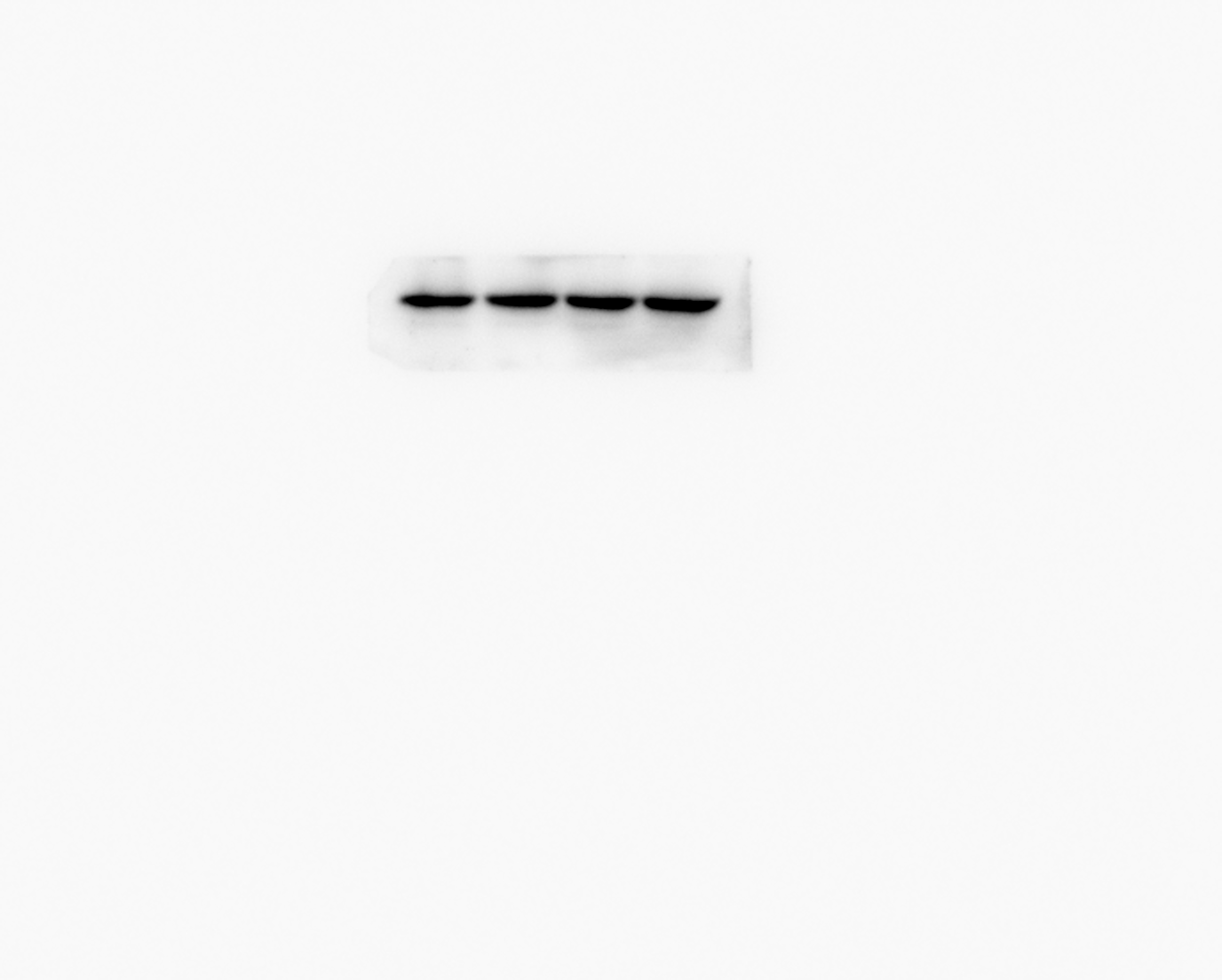


GAPDH (left-to-right):

control,

H/R,

Kae + H/R,

Kae，


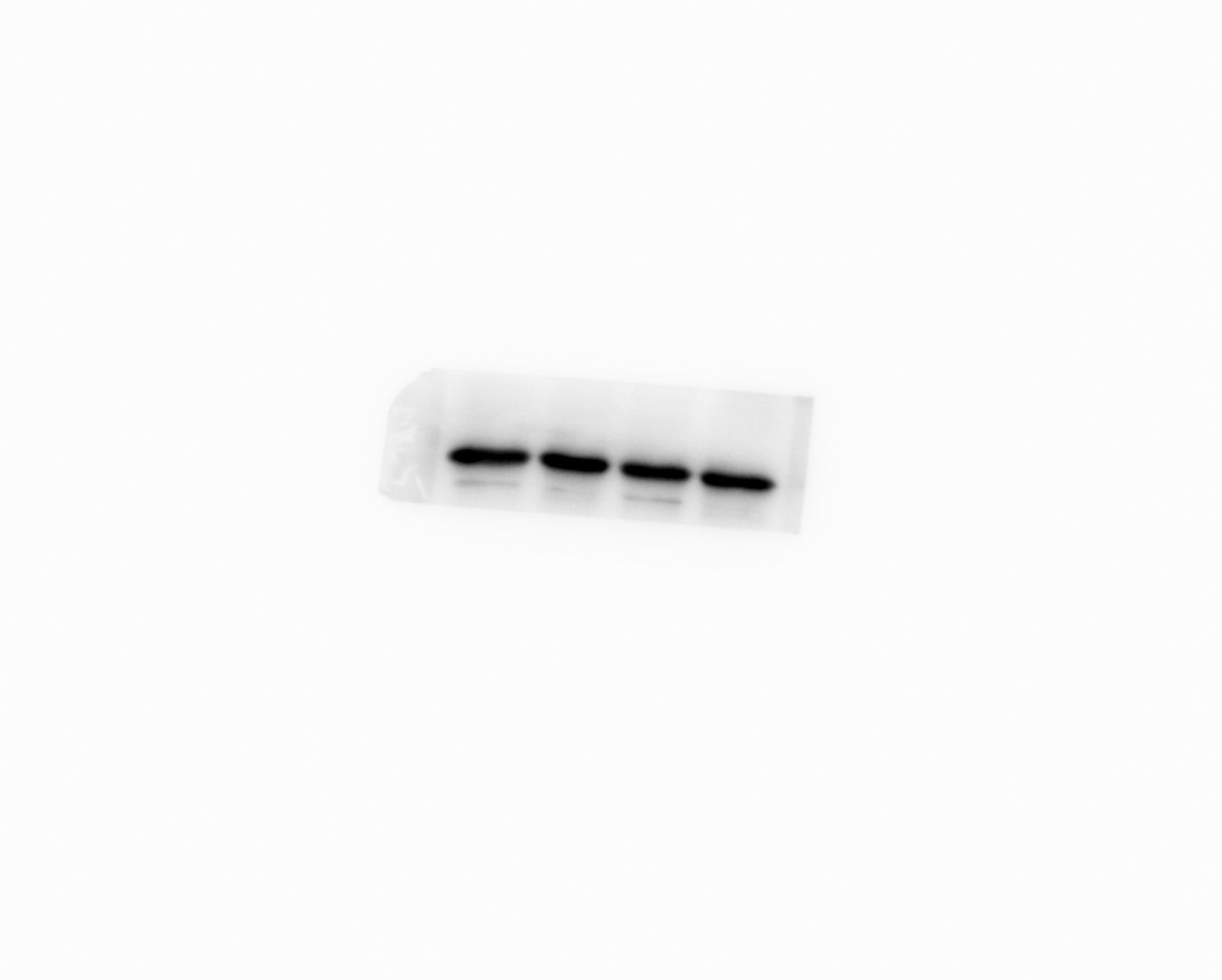


GAPDH (left-to-right):

control,

H/R,

Kae + H/R,

Kae，


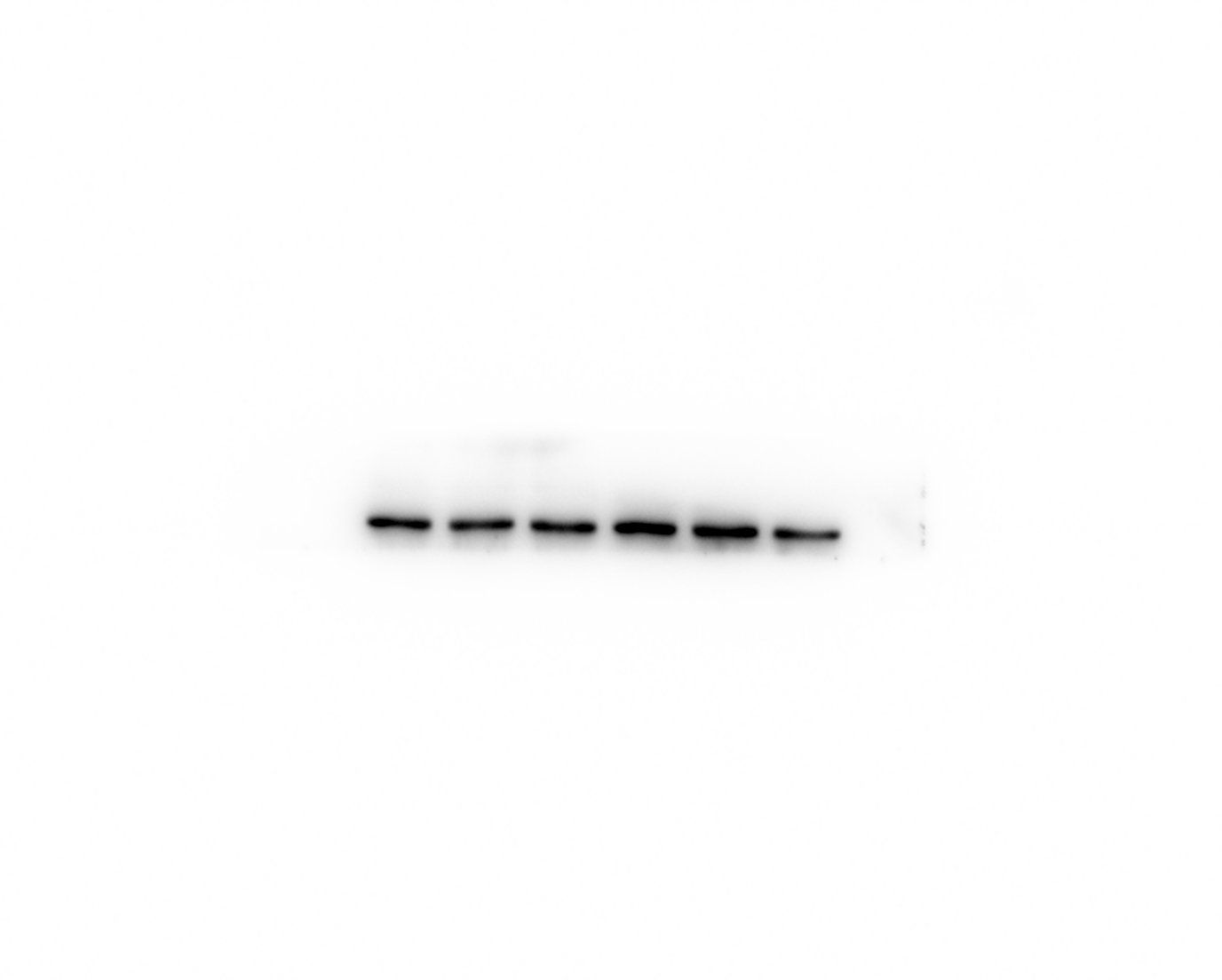


GAPDH (left-to-right):

control,

H/R,

Kae + H/R,

Kae，

Full-length blots/gels are presented in Supplementary Figure 2F

Each experiment was repeated three times

**
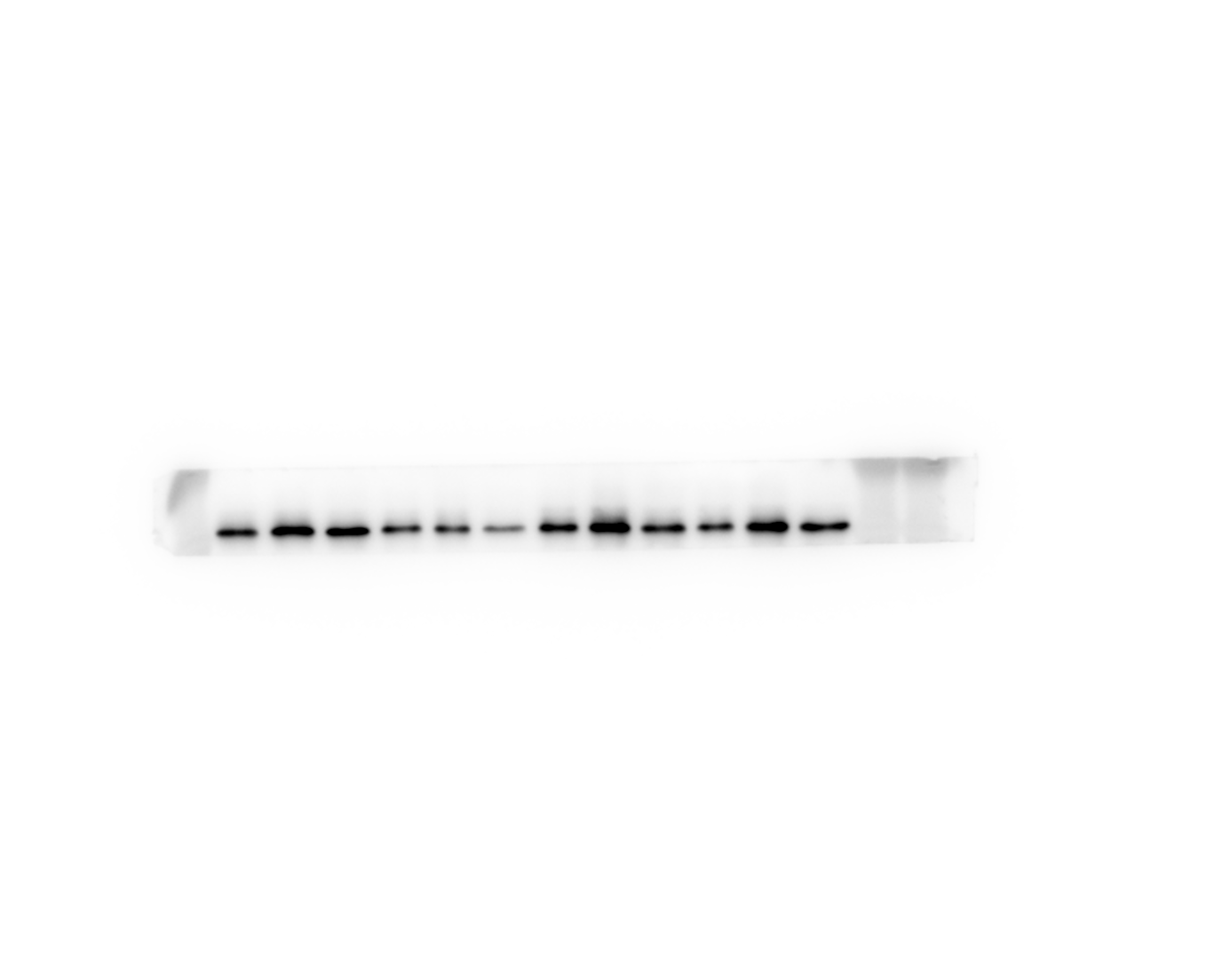
**

Bax (left-to-right):

control,

H/R,

Kae + H/R,

Kae，

marker,

Blank,

control,

H/R,

Kae + H/R,

Kae + H/R + NC,

Kae + H/R + miR-21 I,

miR-21 I,


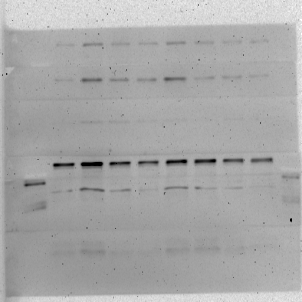


Bax (left-to-right):

control,

H/R,

Kae + H/R,

Kae + H/R + NC,

Kae + H/R + miR-21 I,

miR-21 I,

Blank,

Blank


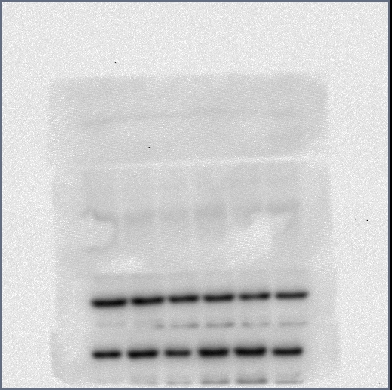


Bax (left-to-right):

control,

H/R,

Kae + H/R,

Kae + H/R + NC,

Kae + H/R + miR-21 I,

miR-21 I,

Blank,

Blank


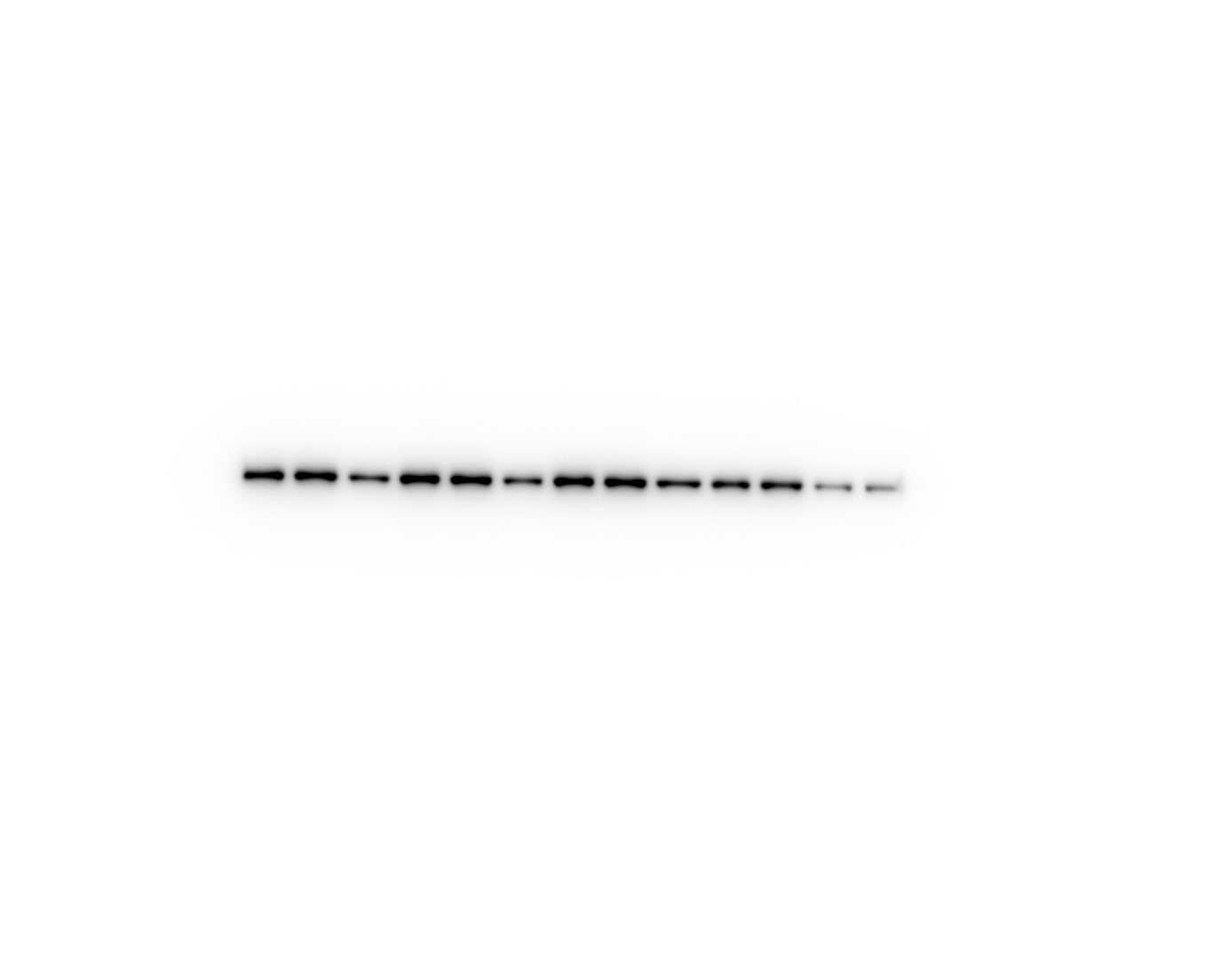


Bcl-2 (left-to-right):

Blank,

blank,

Kae，

Kae + H/R,

H/R,

control,

miR-21 I,

Kae + H/R + miR-21 I,

Kae + H/R + NC,

Kae + H/R,

H/R,

control,


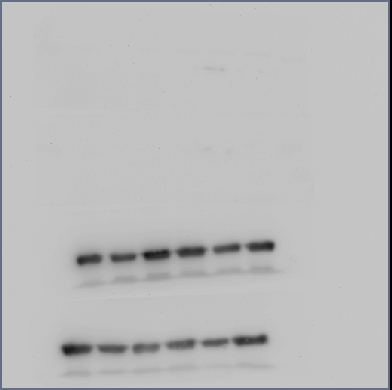
Marker

Bcl-2(left-to-right):

control,

H/R,

Kae + H/R,

Kae + H/R + NC,

Kae + H/R + miR-21 I,

miR-21 I,


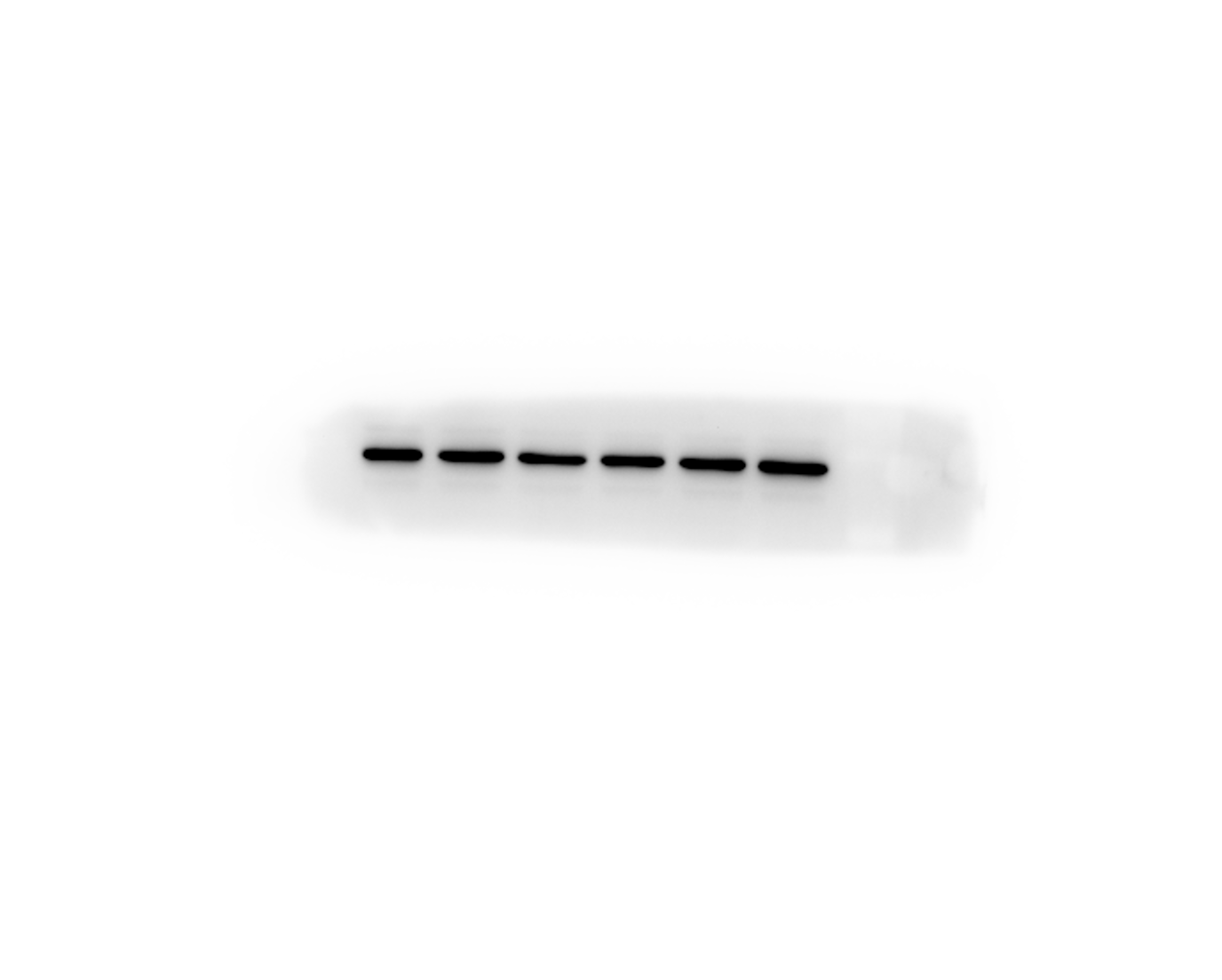


GAPDH (left-to-right):

control,

H/R,

Kae + H/R,

Kae + H/R + NC,

Kae + H/R + miR-21 I,

miR-21 I,


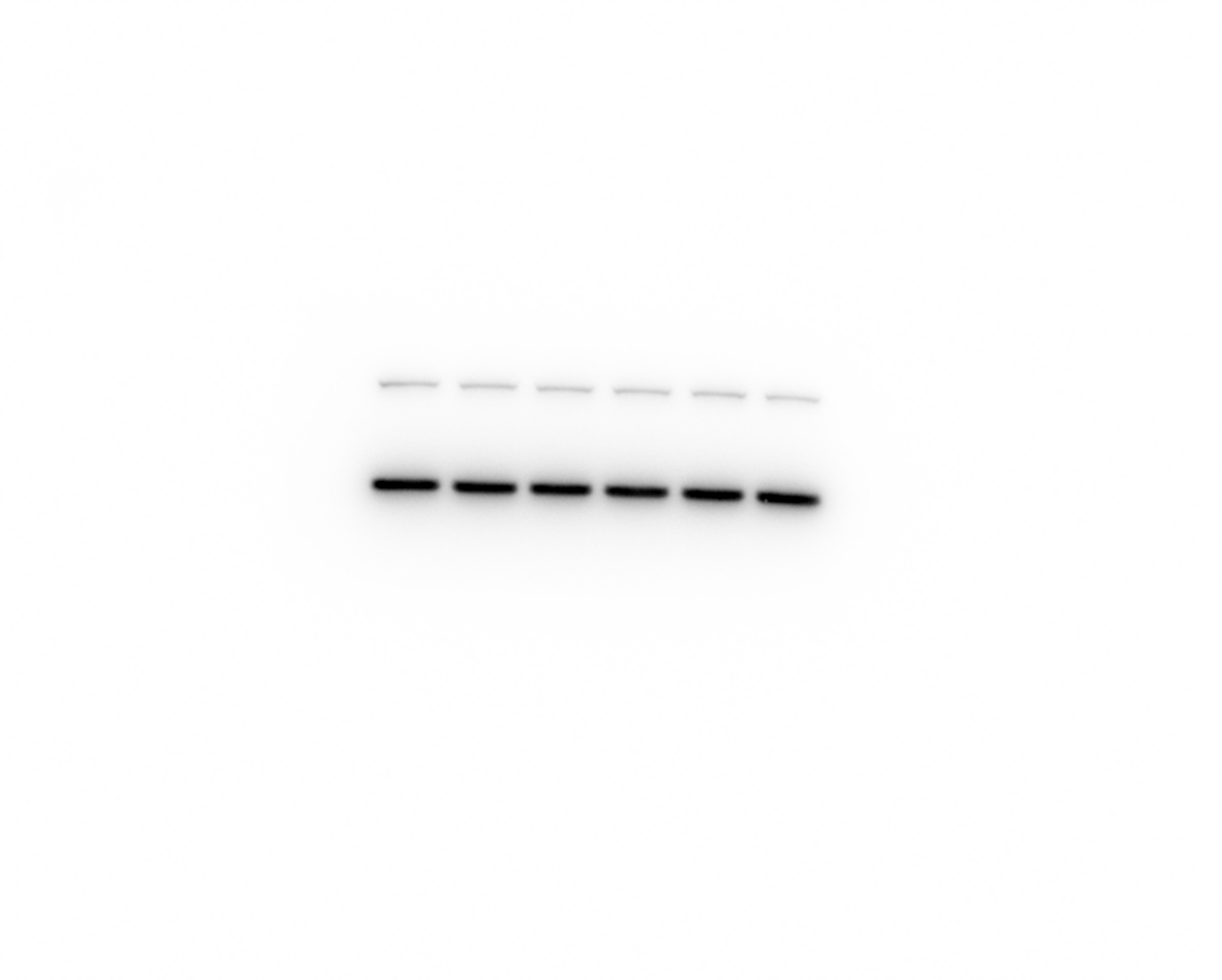


GAPDH (left-to-right):

control,

H/R,

Kae + H/R,

Kae + H/R + NC,

Kae + H/R + miR-21 I,

miR-21 I,


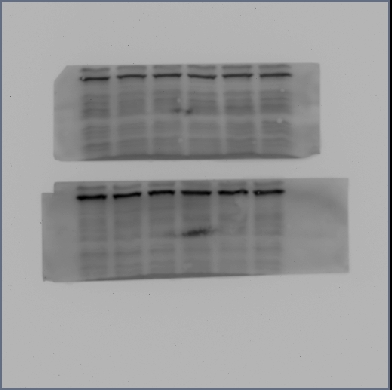


GAPDH (left-to-right):

control,

H/R,

Kae + H/R,

Kae + H/R + NC,

Kae + H/R + miR-21 I,

miR-21 I,

Full-length blots/gels are presented in Supplementary Figure 5A

Each experiment was repeated three times


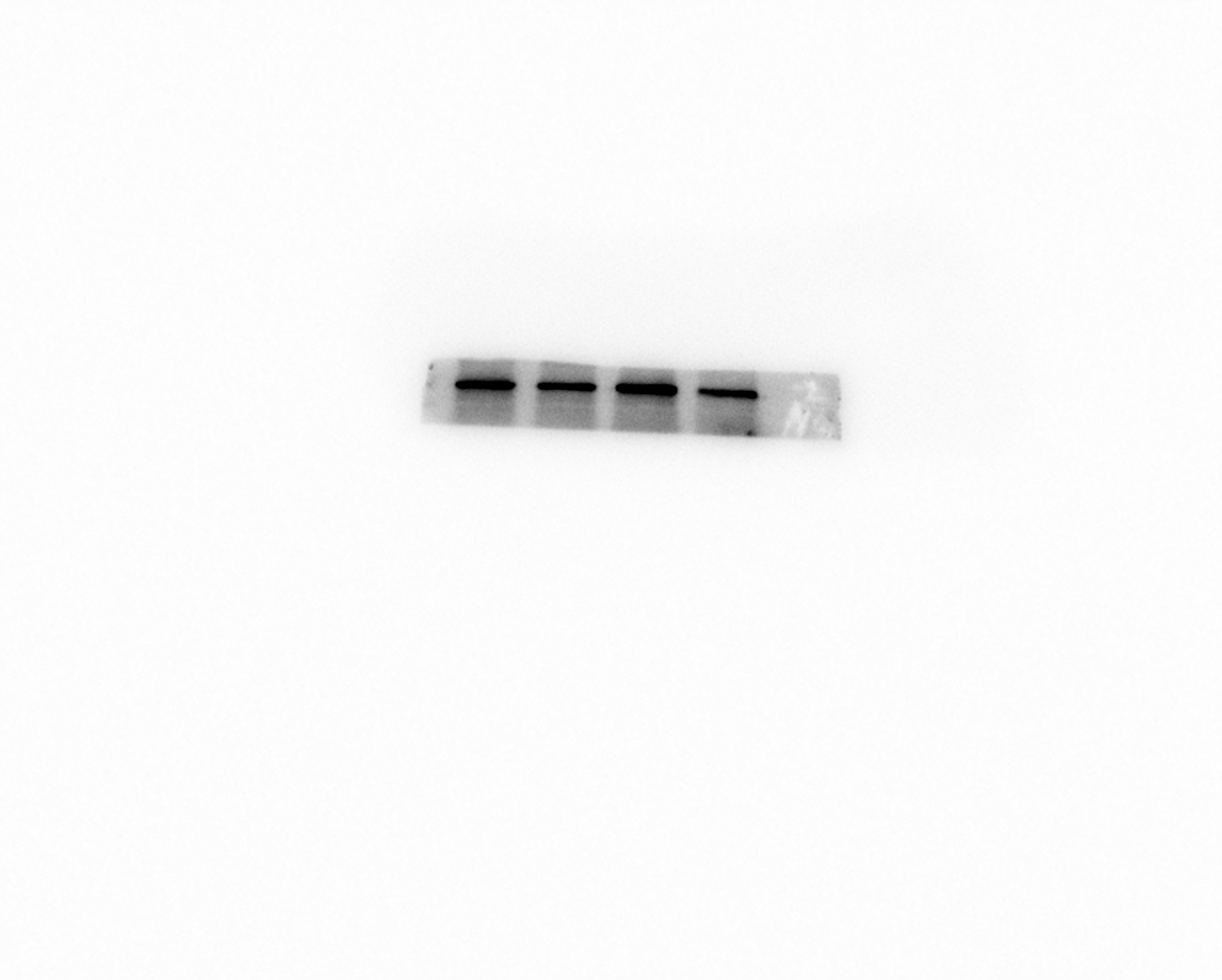


Nocth 1 (left-to-right):

control,

H/R,

Kae + H/R,

Kae，


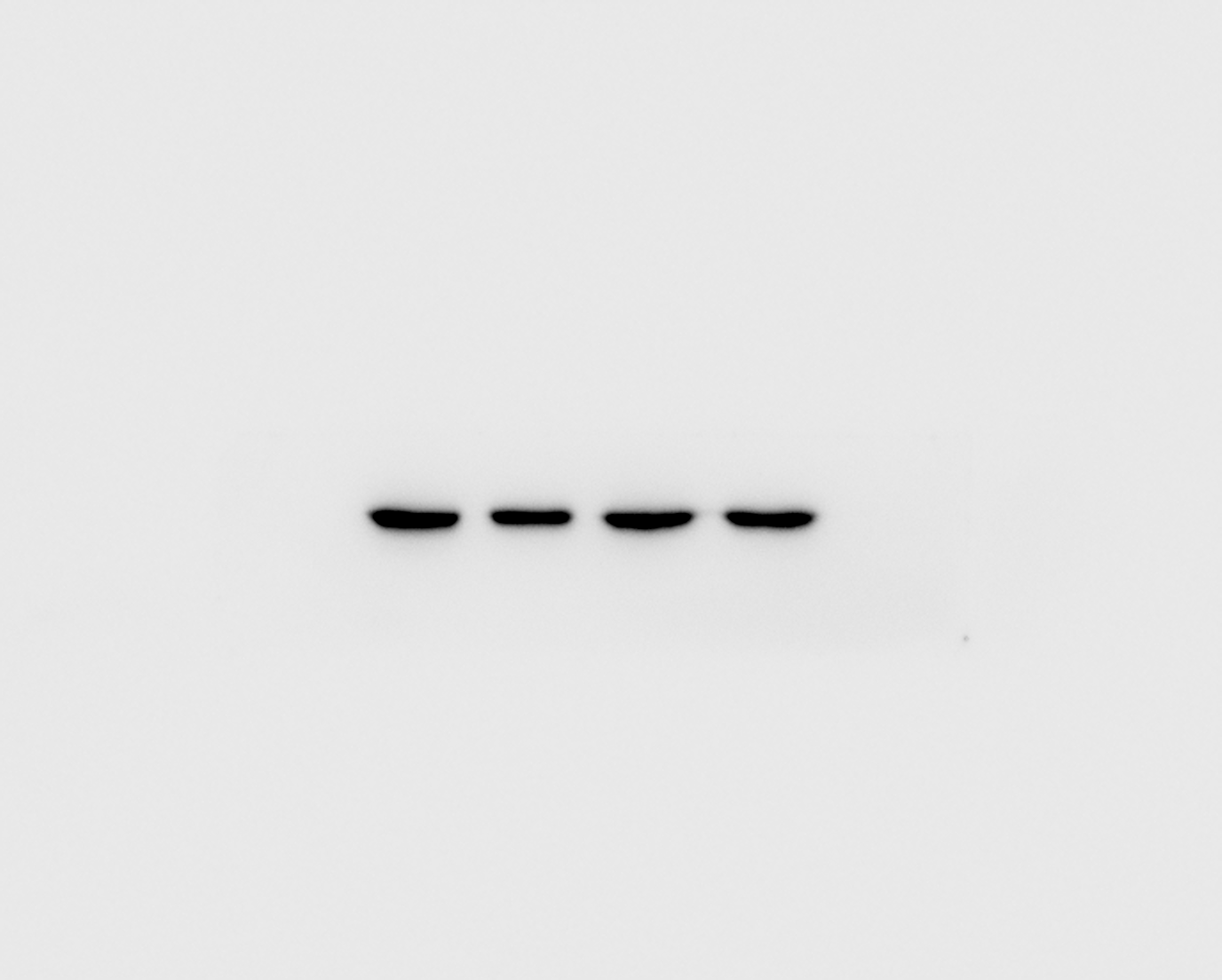


Notch1 (left-to-right):

control,

H/R,

Kae + H/R,

Kae，


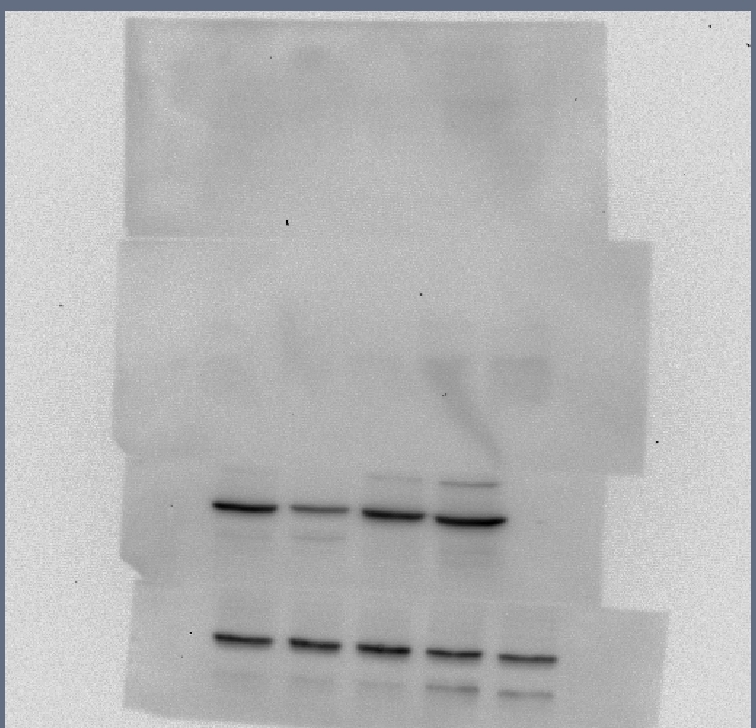


Notch1(left-to-right):

control,

H/R,

Kae + H/R,

Kae，


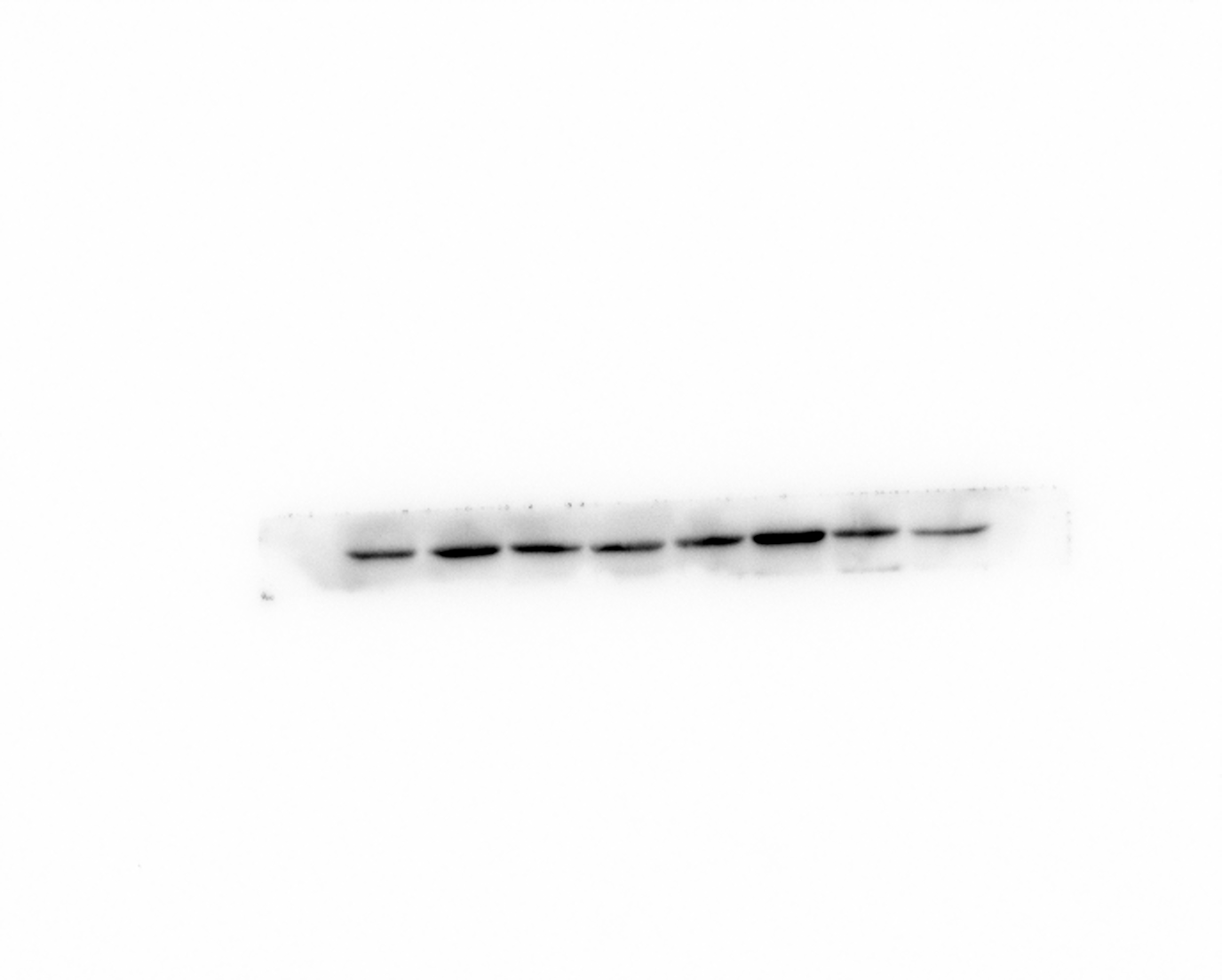


PTEN (left-to-right):

control,

H/R,

Kae + H/R,

Kae，

control,

H/R,

Kae + H/R,

Kae，


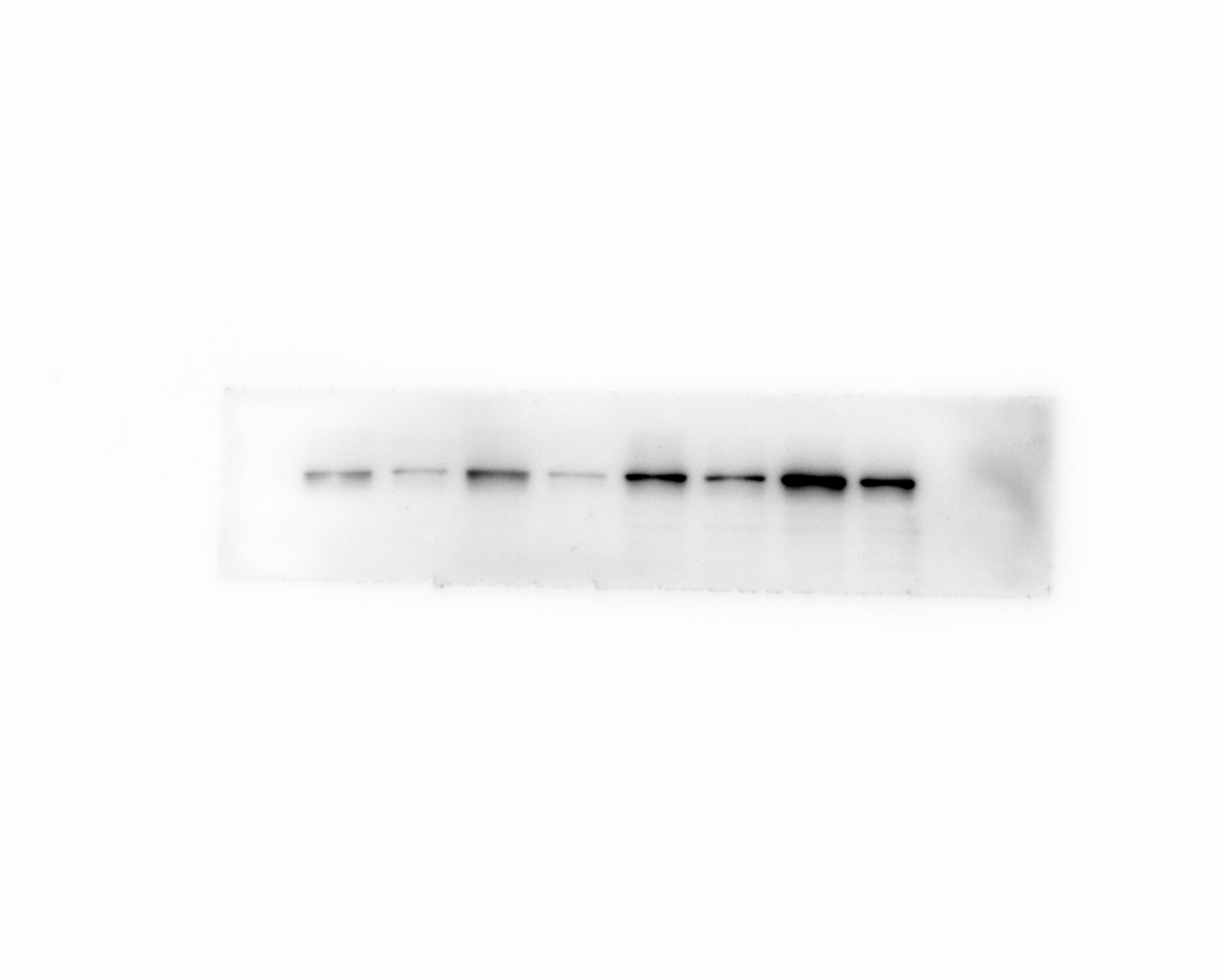


PTEN (left-to-right):

control,

H/R,

Kae + H/R,

Kae，

control,

H/R,

Kae + H/R,

Kae，


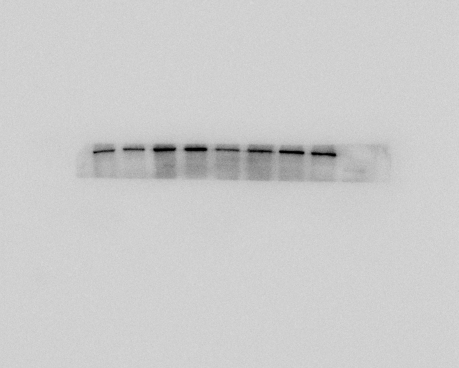


P-AKT (left-to-right):

control,

H/R,

Kae + H/R,

Kae，

control,

H/R,

Kae + H/R,

Kae，


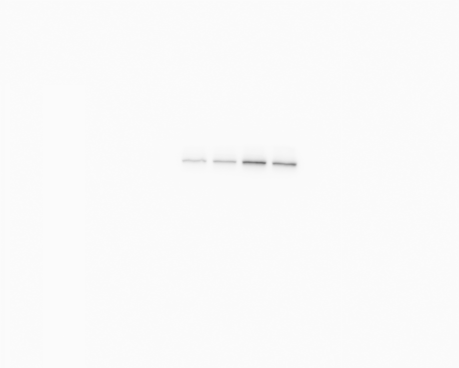


P-AKT (left-to-right):

control,

H/R,

Kae + H/R,

Kae


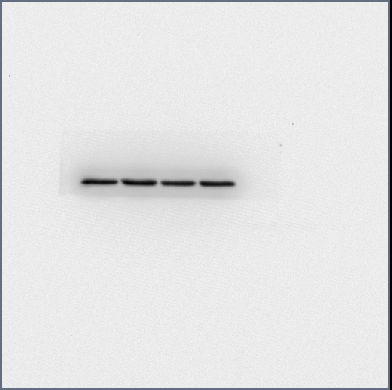


AKT (left-to-right):

control,

H/R,

Kae + H/R,

Kae


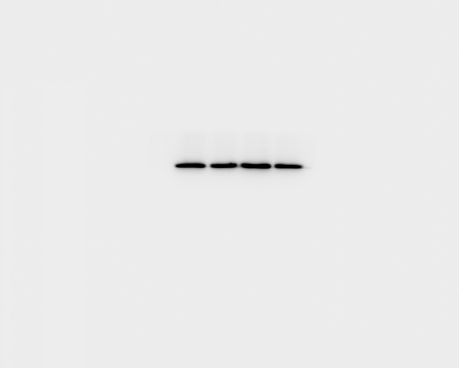


AKT (left-to-right):

control,

H/R,

Kae + H/R,

Kae


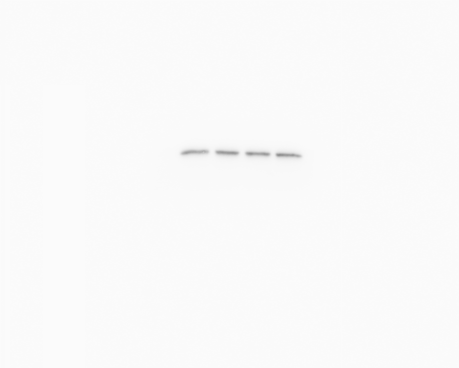


AKT (left-to-right):

control,

H/R,

Kae + H/R,

Kae


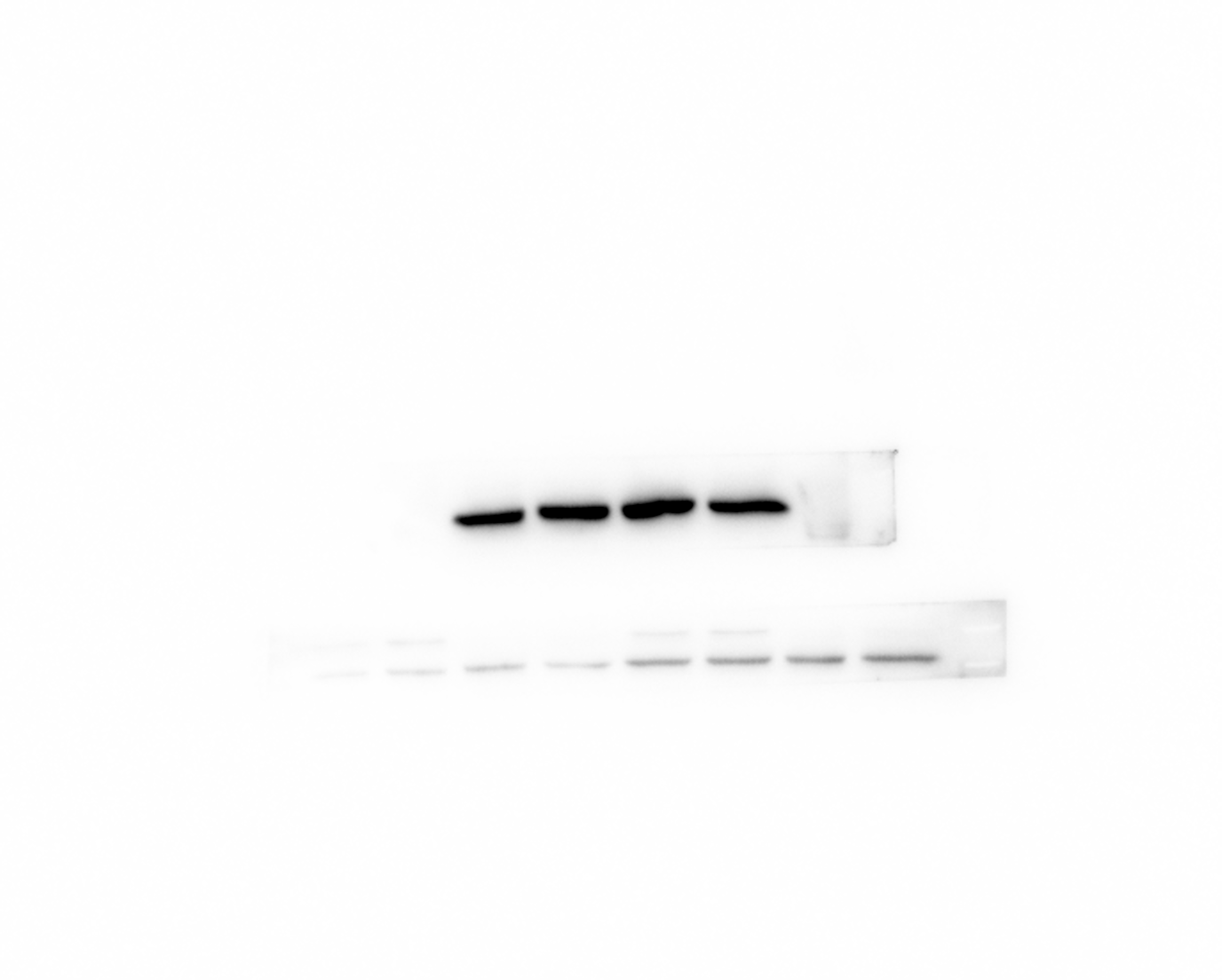


GAPDH (left-to-right):

control,

H/R,

Kae + H/R,

Kae


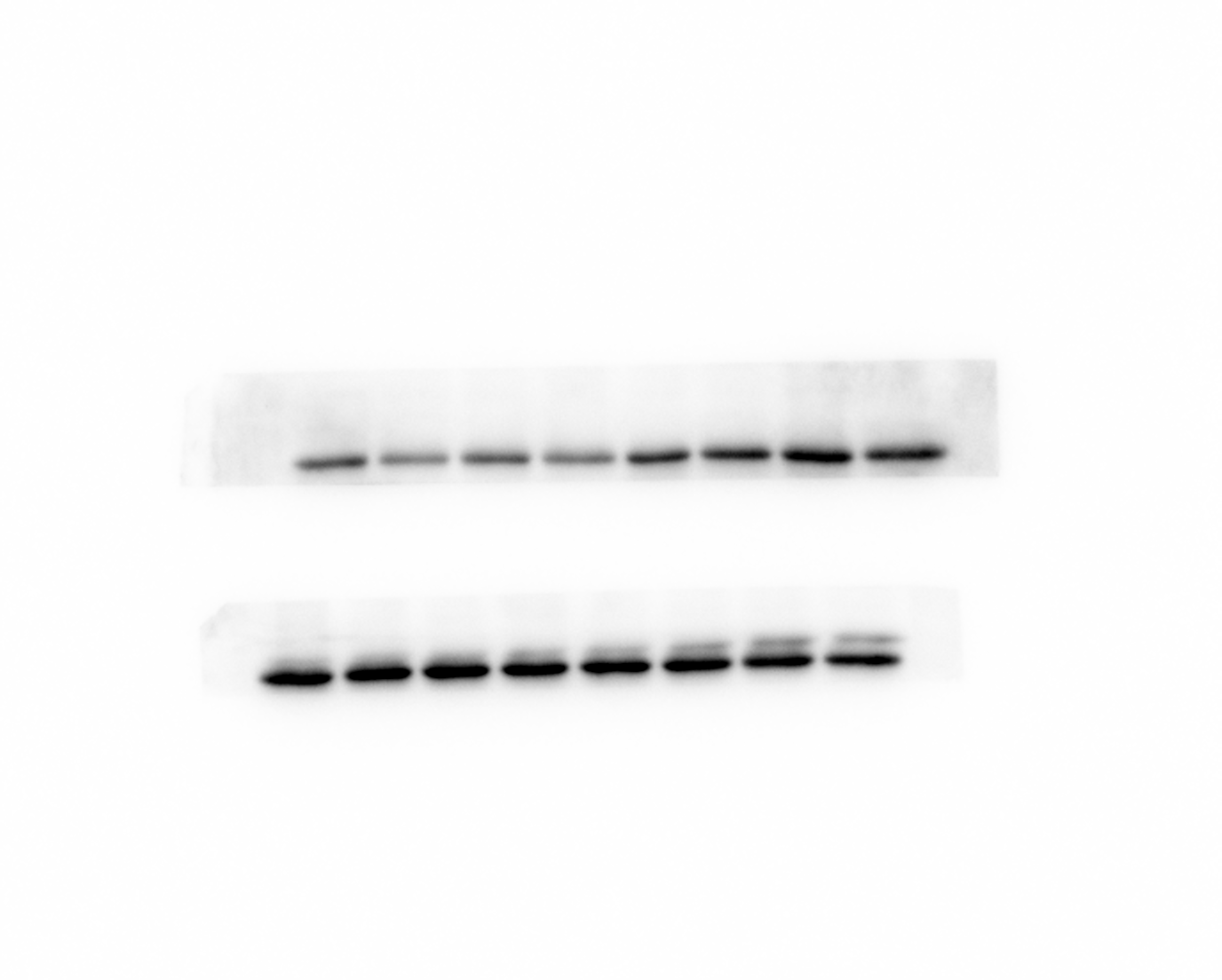


GAPDH (left-to-right):

control,

H/R,

Kae + H/R,

Kae，

control,

H/R,

Kae + H/R,

Kae，

Full-length blots/gels are presented in Supplementary Figure 5E

Each experiment was repeated three times


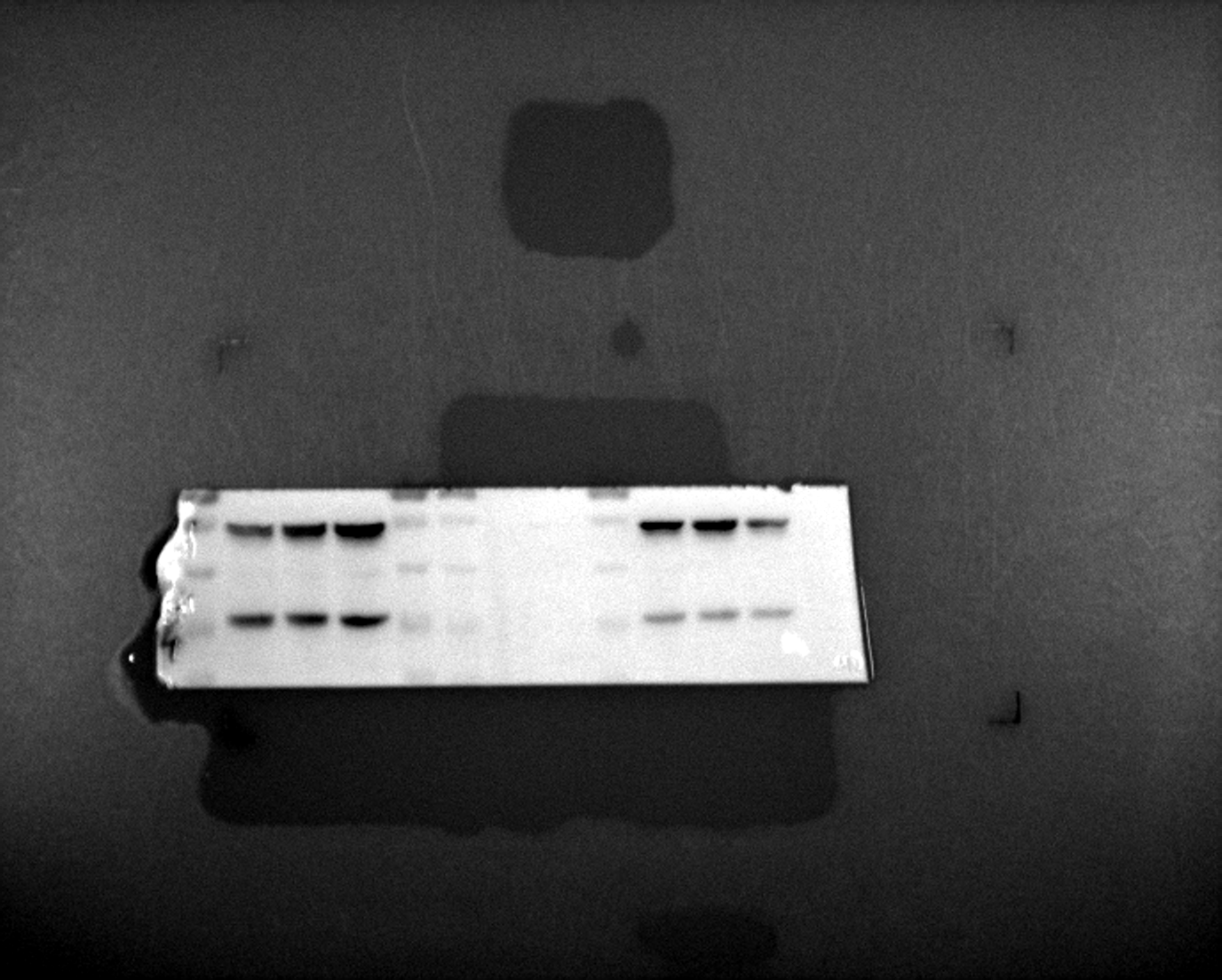


Nocth-1 (left-to-right):

Nocth 1 siRNA,

Control siRNA,

control,

Blank,

control,

Control siRNA,

Nocth 1 siRNA,


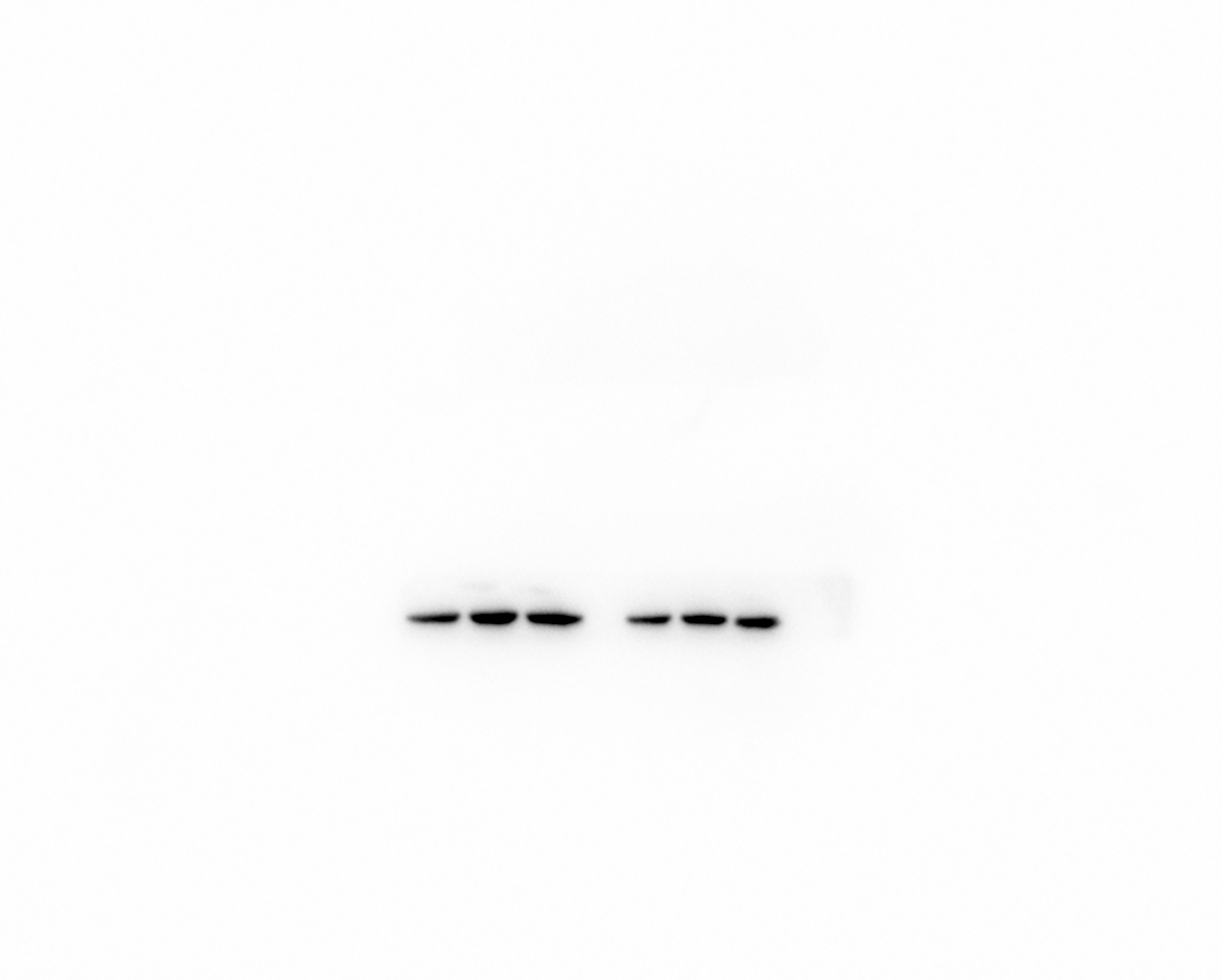


Nocth-1 (left-to-right):

control,

Control siRNA,

Nocth 1 siRNA,

Blank,

control,

Control siRNA,

Nocth 1 siRNA,


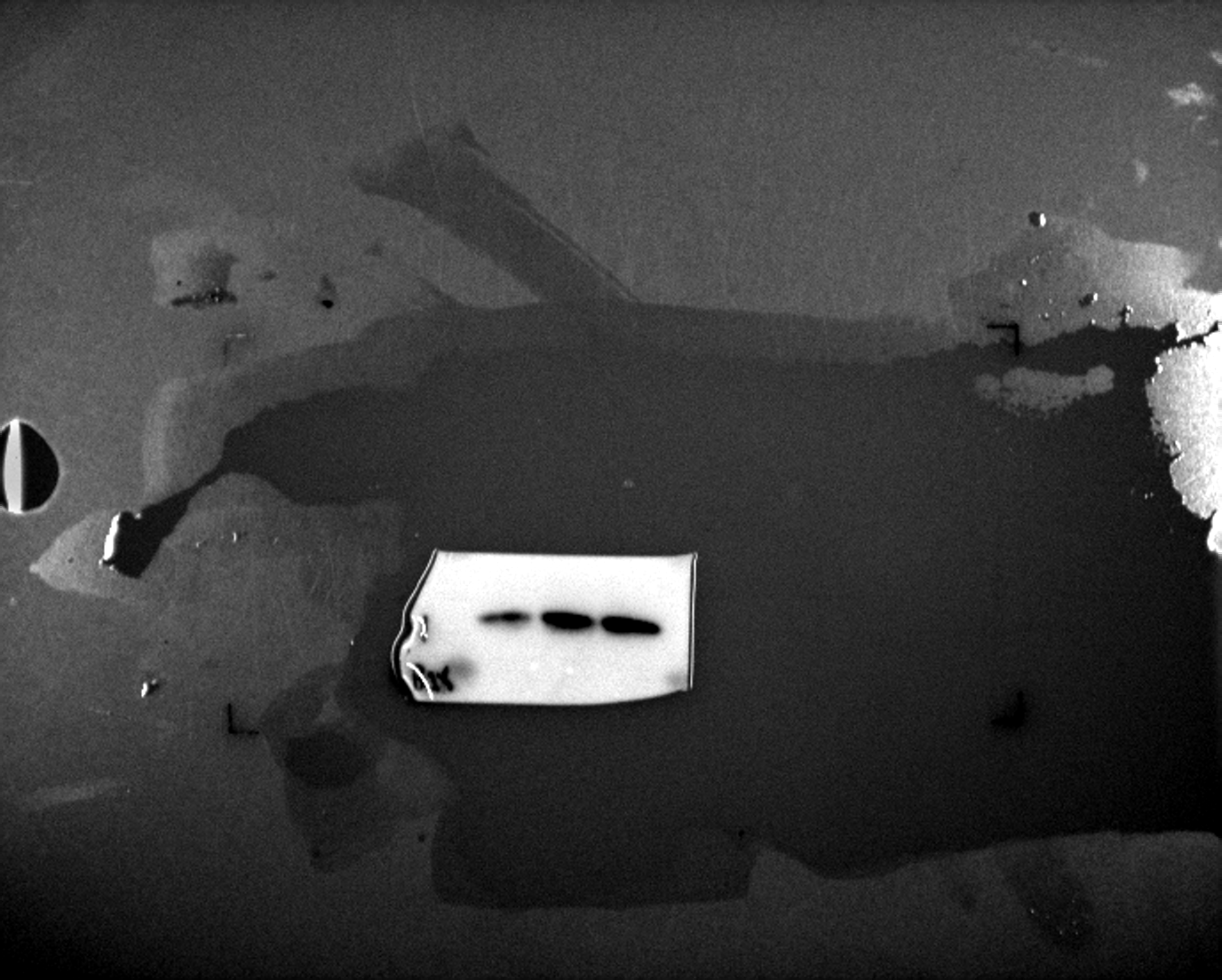


Nocth-1 (left-to-right):

control,

Control siRNA,

Nocth 1 siRNA,


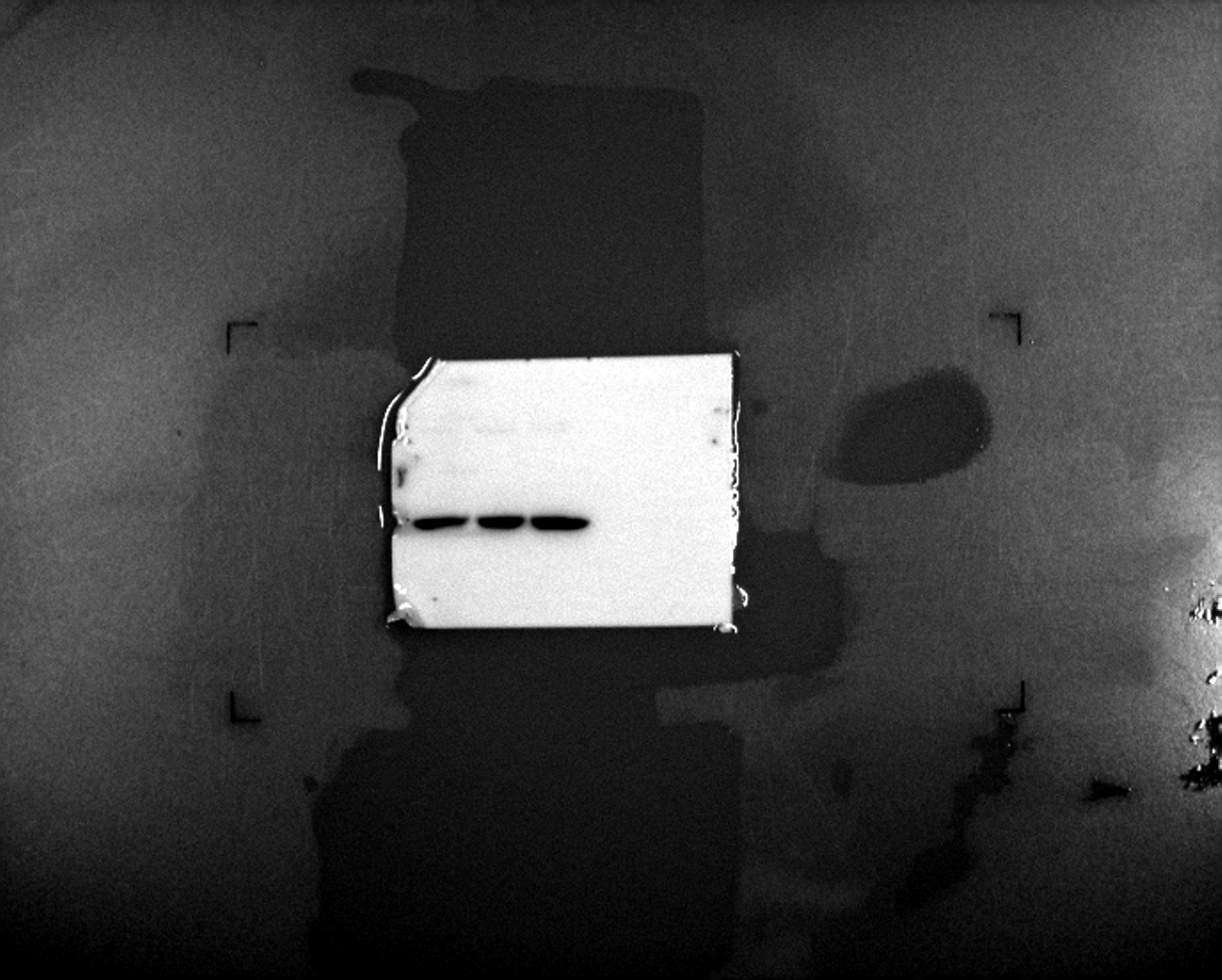


GAPDH (left-to-right):

control,

Control siRNA,

Nocth 1 siRNA,


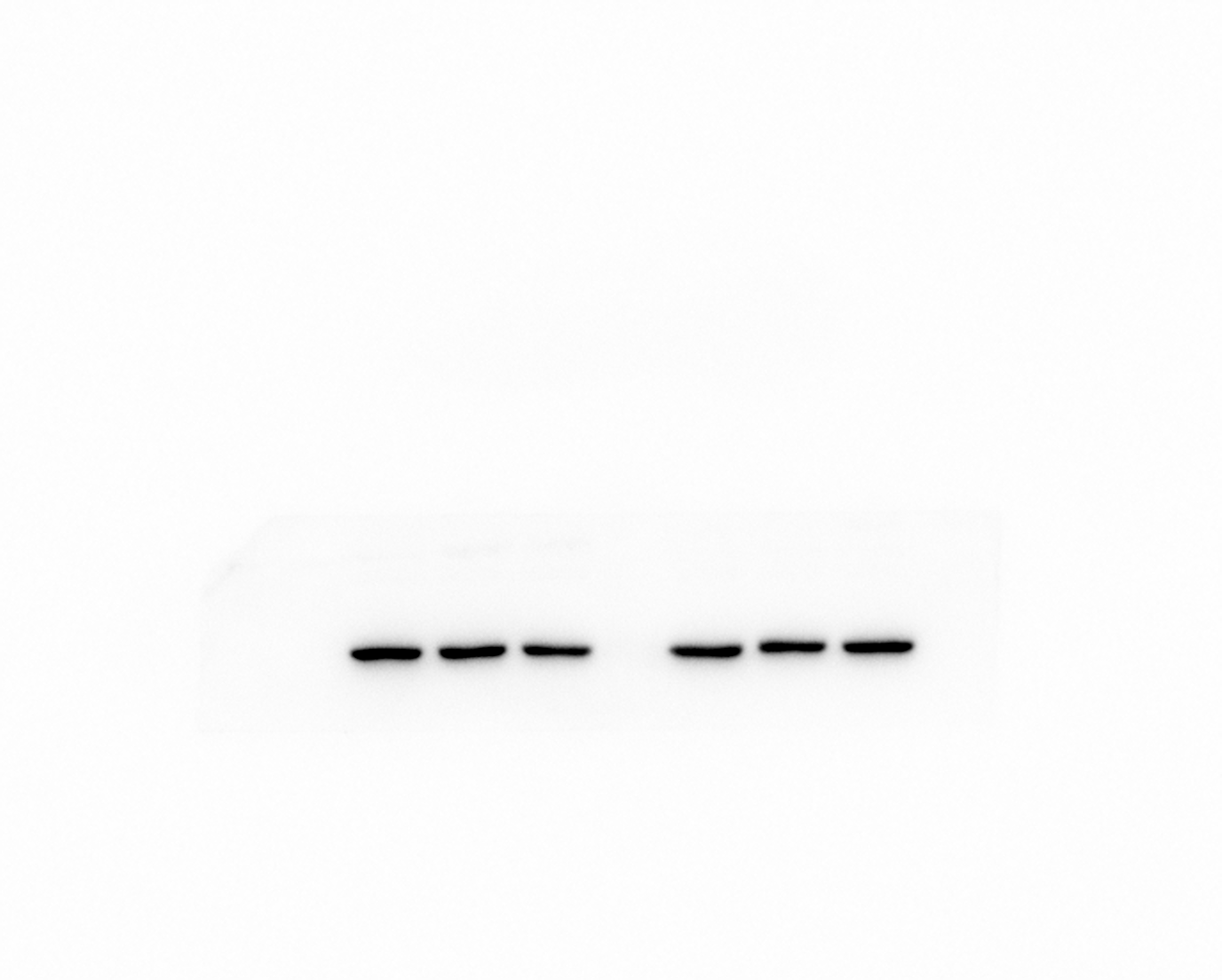


Nocth-1 (left-to-right):

control,

Control siRNA,

Nocth 1 siRNA,

Blank,

control,

Control siRNA,

Nocth 1 siRNA,


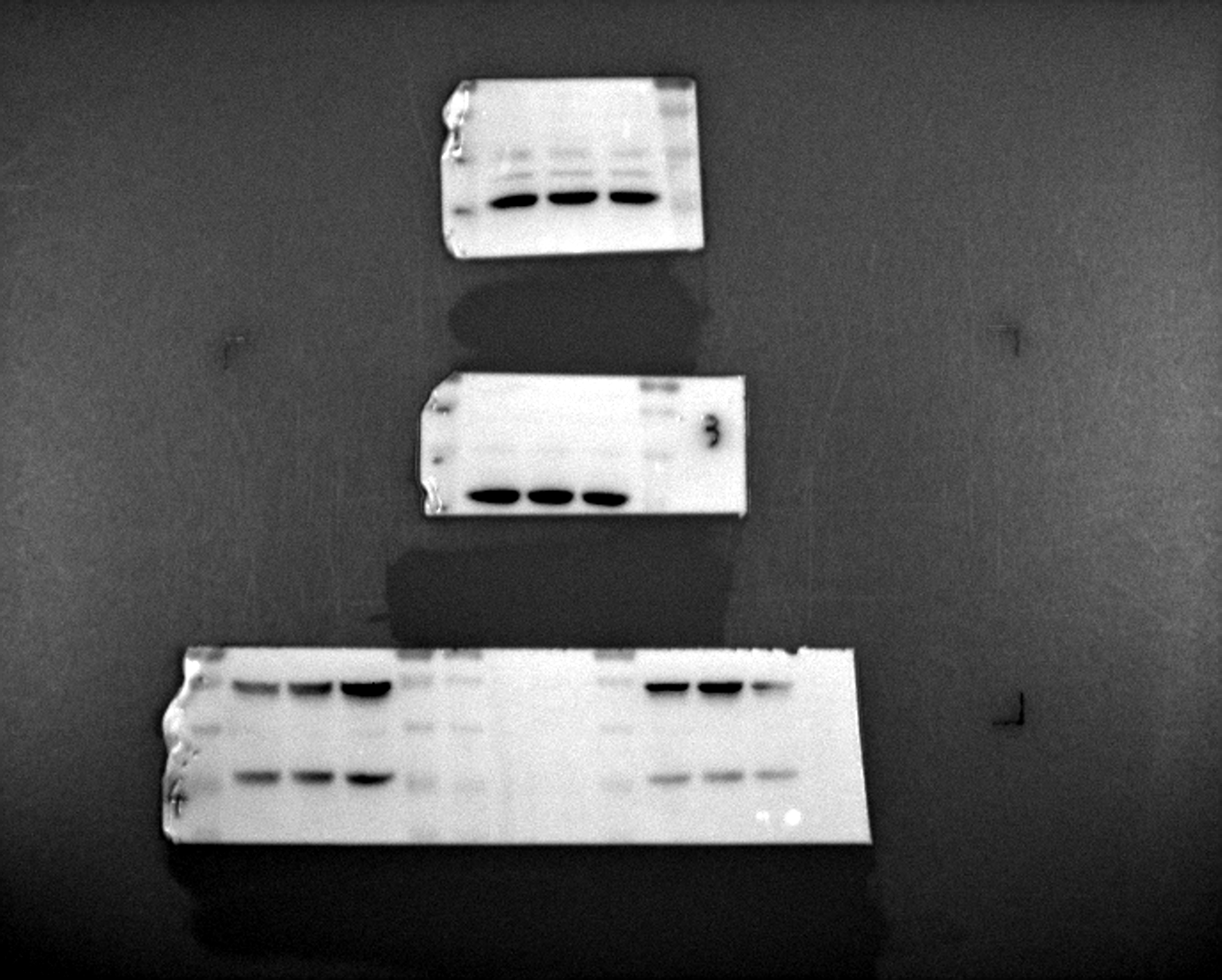


Nocth-1 (left-to-right):

control,

Control siRNA,

Nocth 1 siRNA,

Full-length blots/gels are presented in Supplementary Figure A

Each experiment was repeated three times


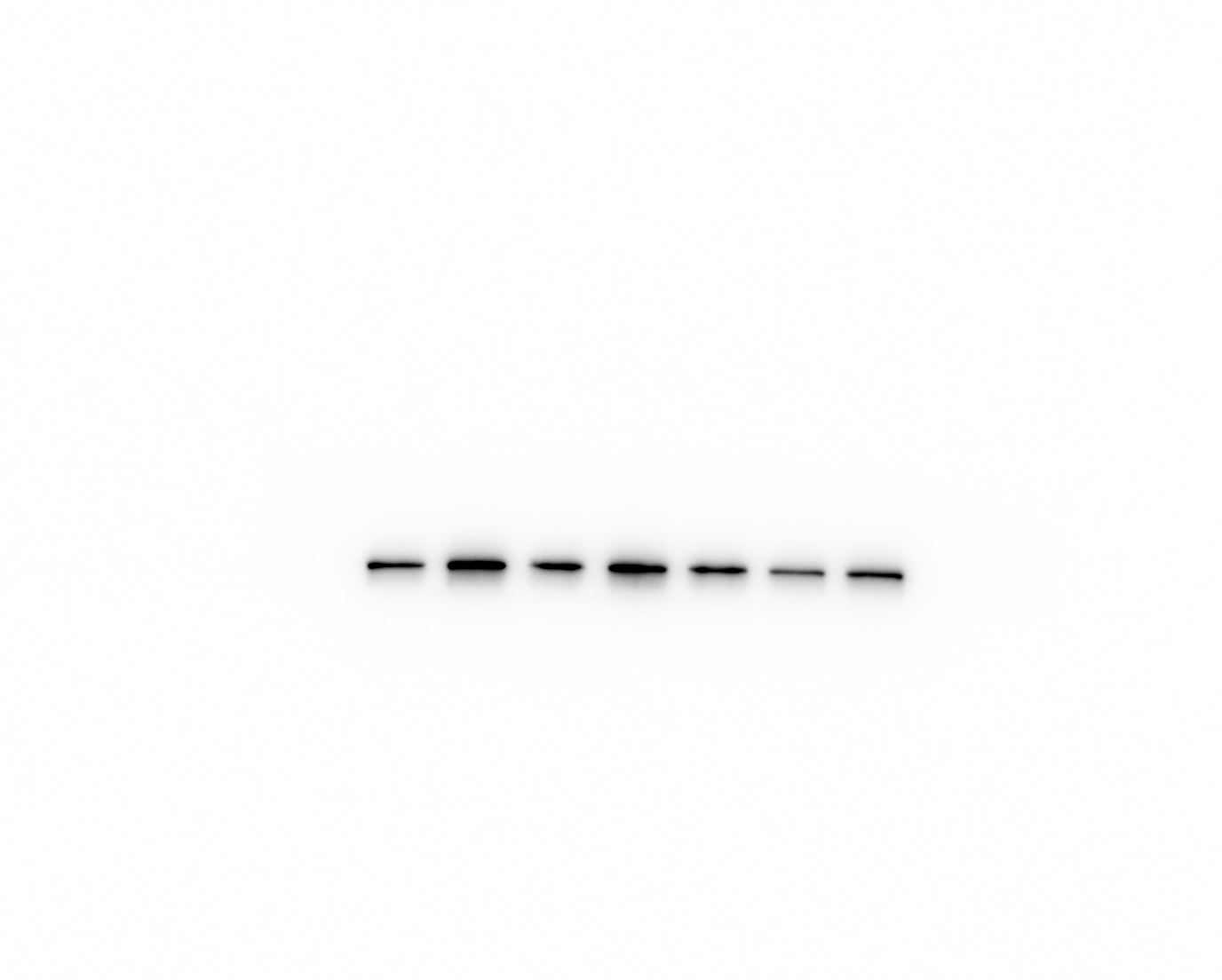


Notch-1 (left-to-right):

Marker,

control,

H/R,

Kae + H/R,

Kae + H/R + NC,

Kae + H/R + miR-21 I,

miR-21 I,


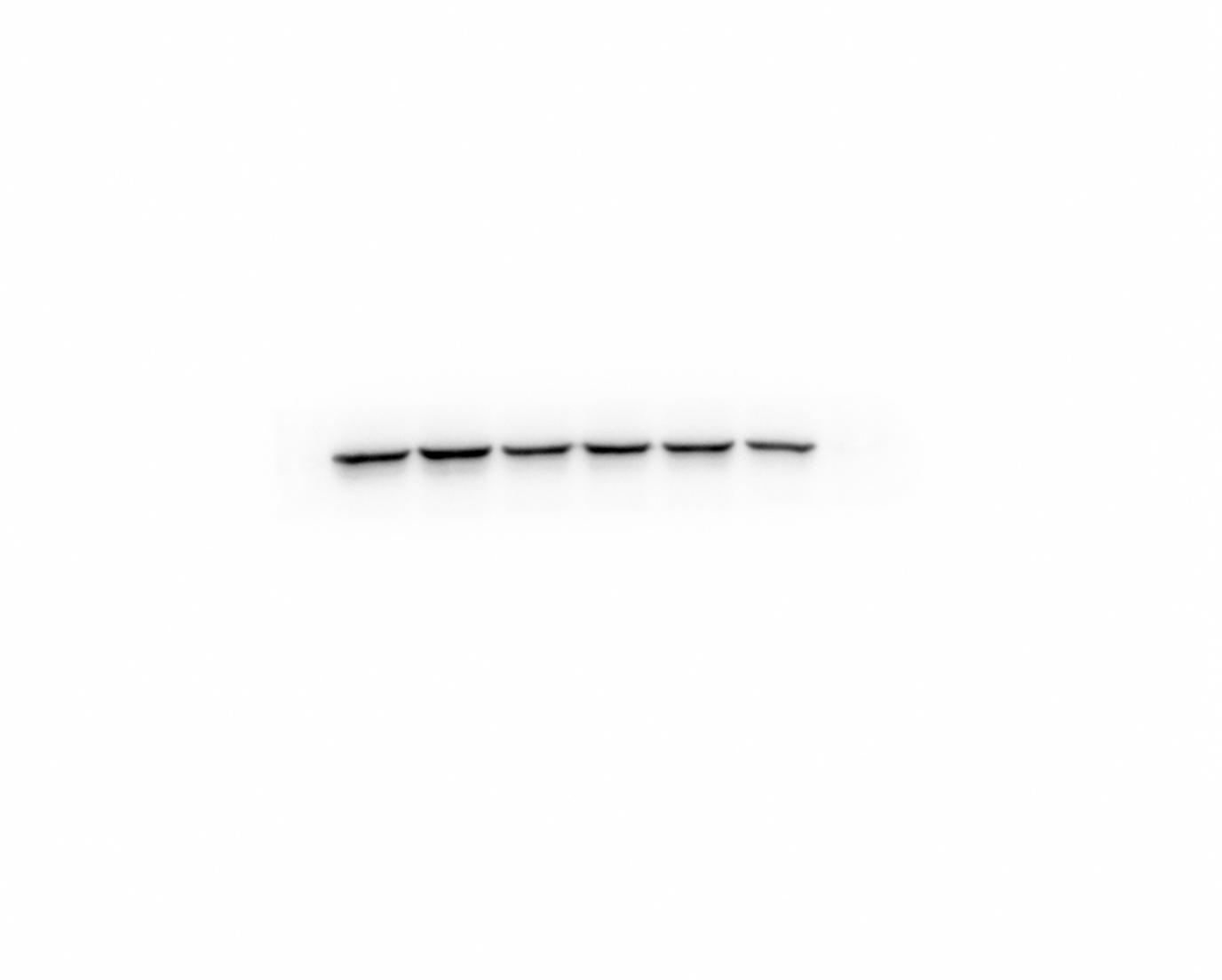


PTEN (left-to-right):

control,

H/R,

Kae + H/R,

Kae + H/R + NC,

Kae + H/R + miR-21 I,

miR-21 I,


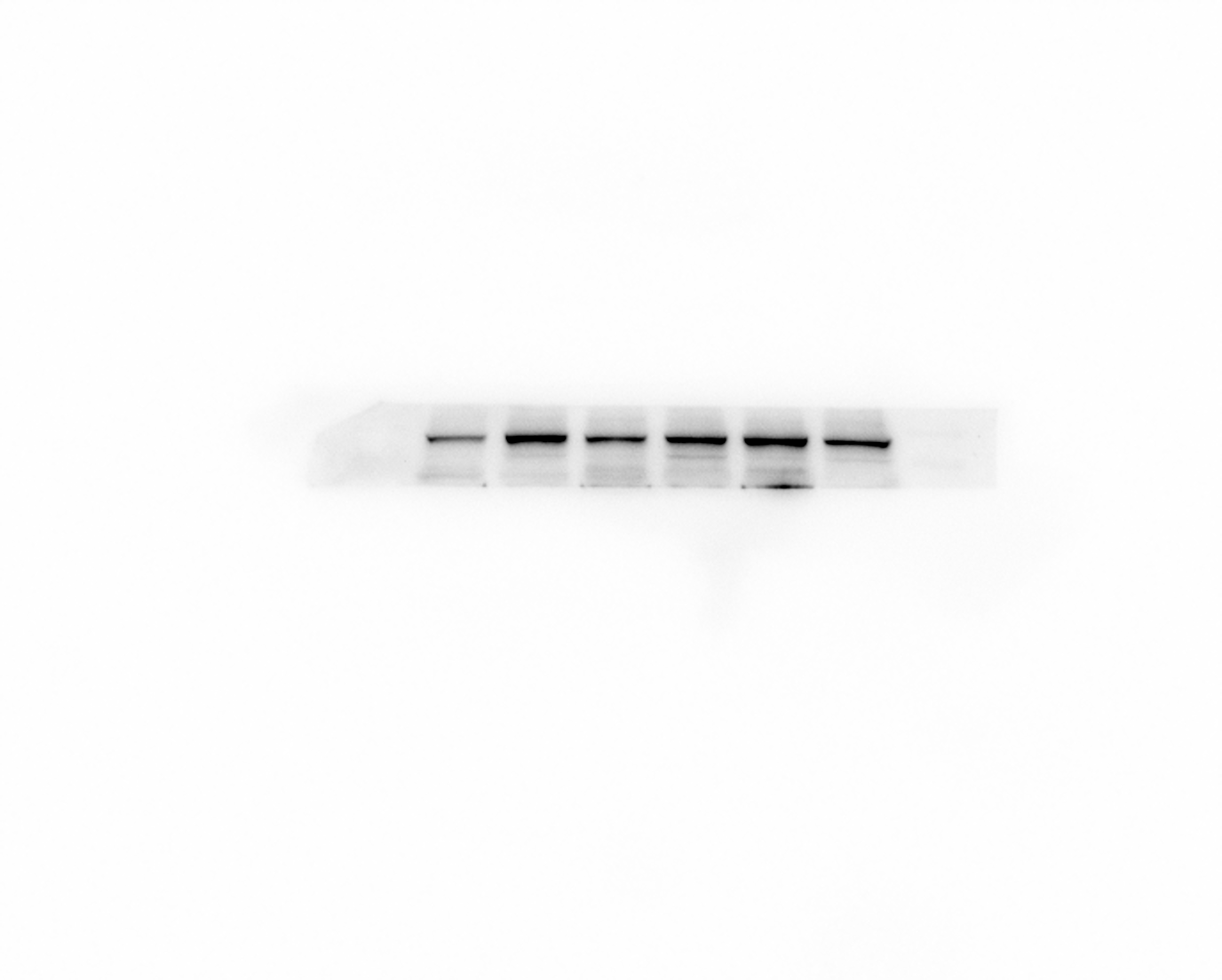


PTEN (left-to-right):

control,

H/R,

Kae + H/R,

Kae + H/R + NC,

Kae + H/R + miR-21 I,

miR-21 I,


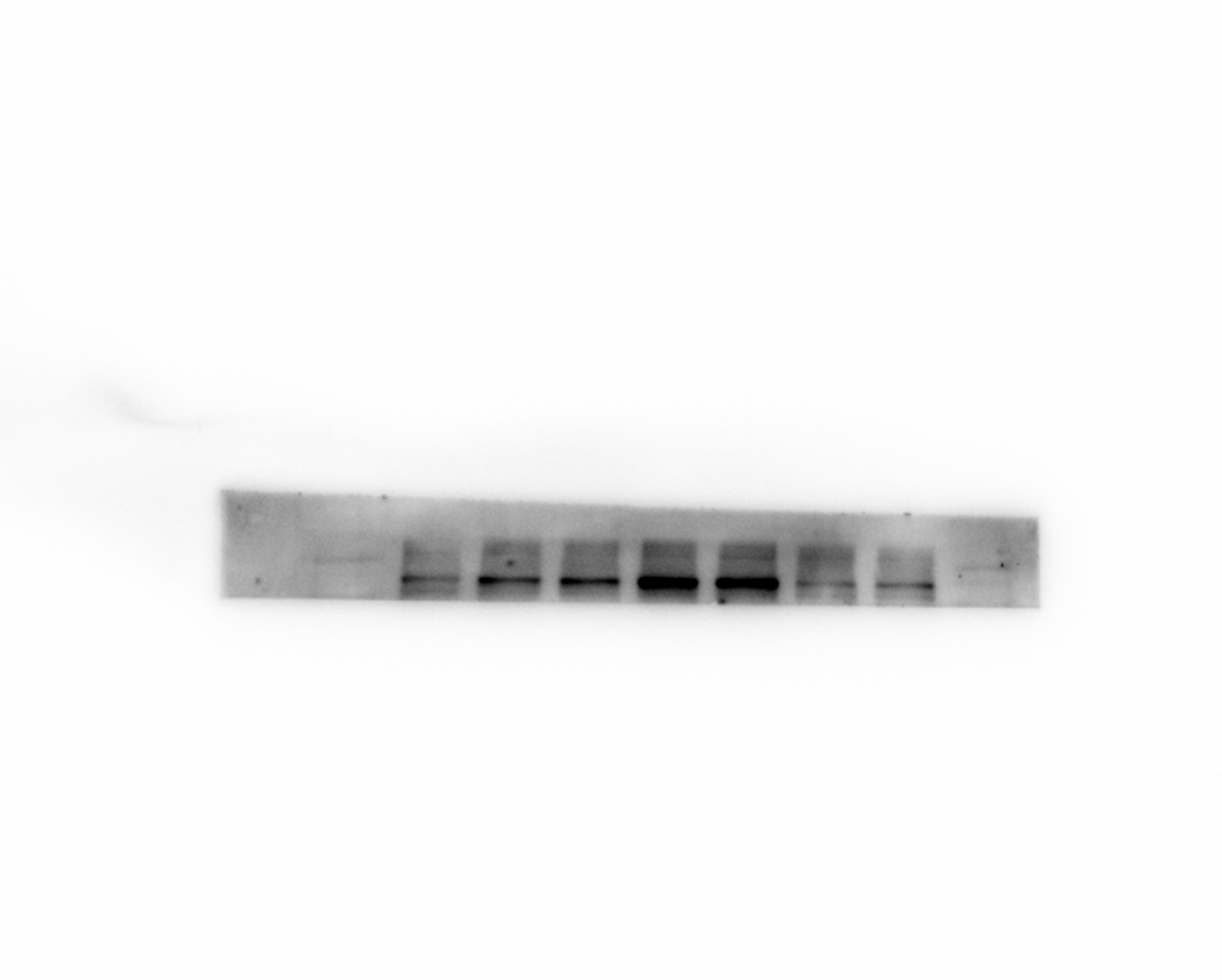
P-AKT (left-to-right):

Marker,

control,

H/R,

Kae + H/R,

Kae + H/R + NC,

Kae + H/R + miR-21 I,

miR-21 I,


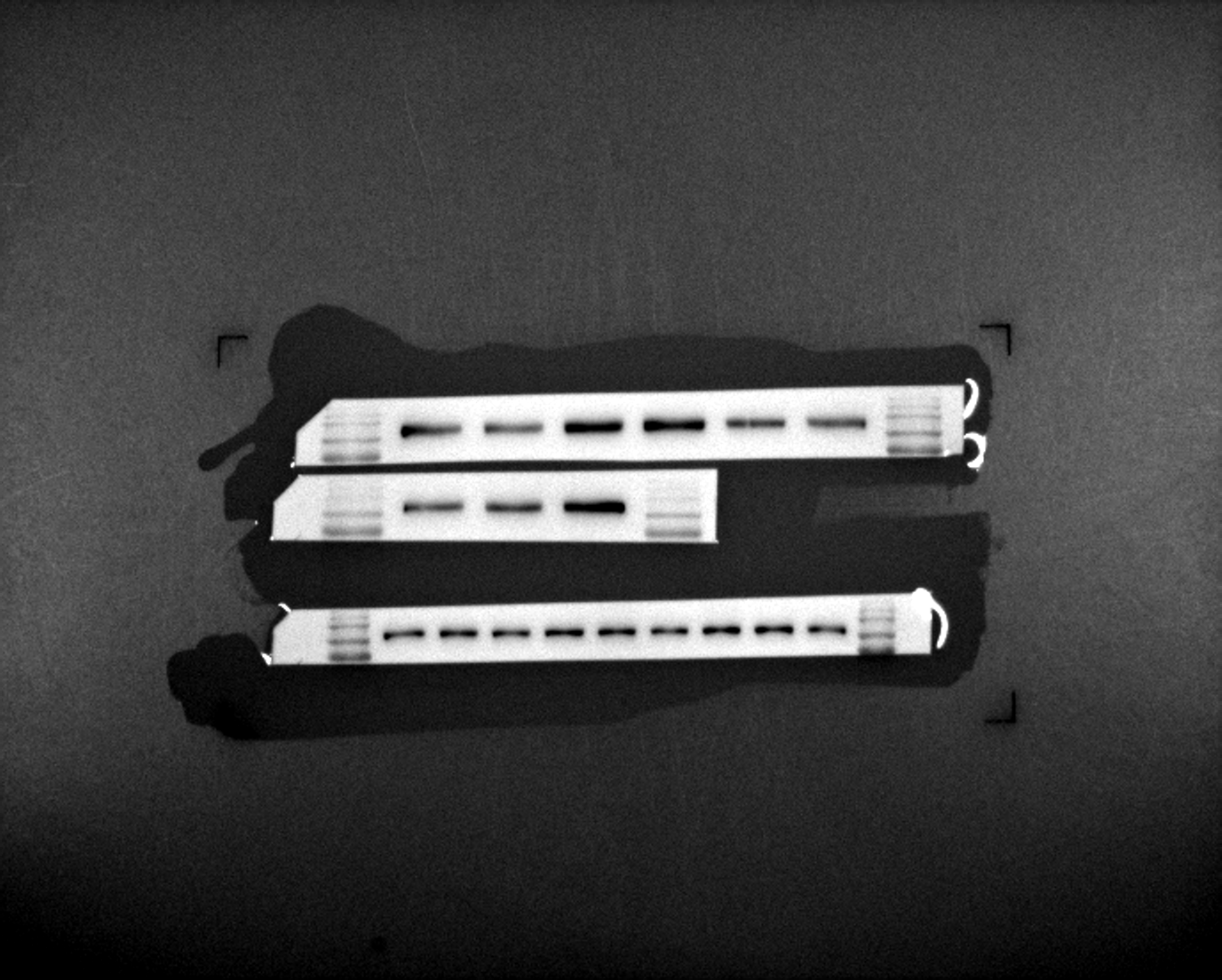


P-AKT (left-to-right):

Marker,

control,

H/R,

Kae + H/R,

Kae + H/R + NC,

Kae + H/R + miR-21 I,

miR-21 I,


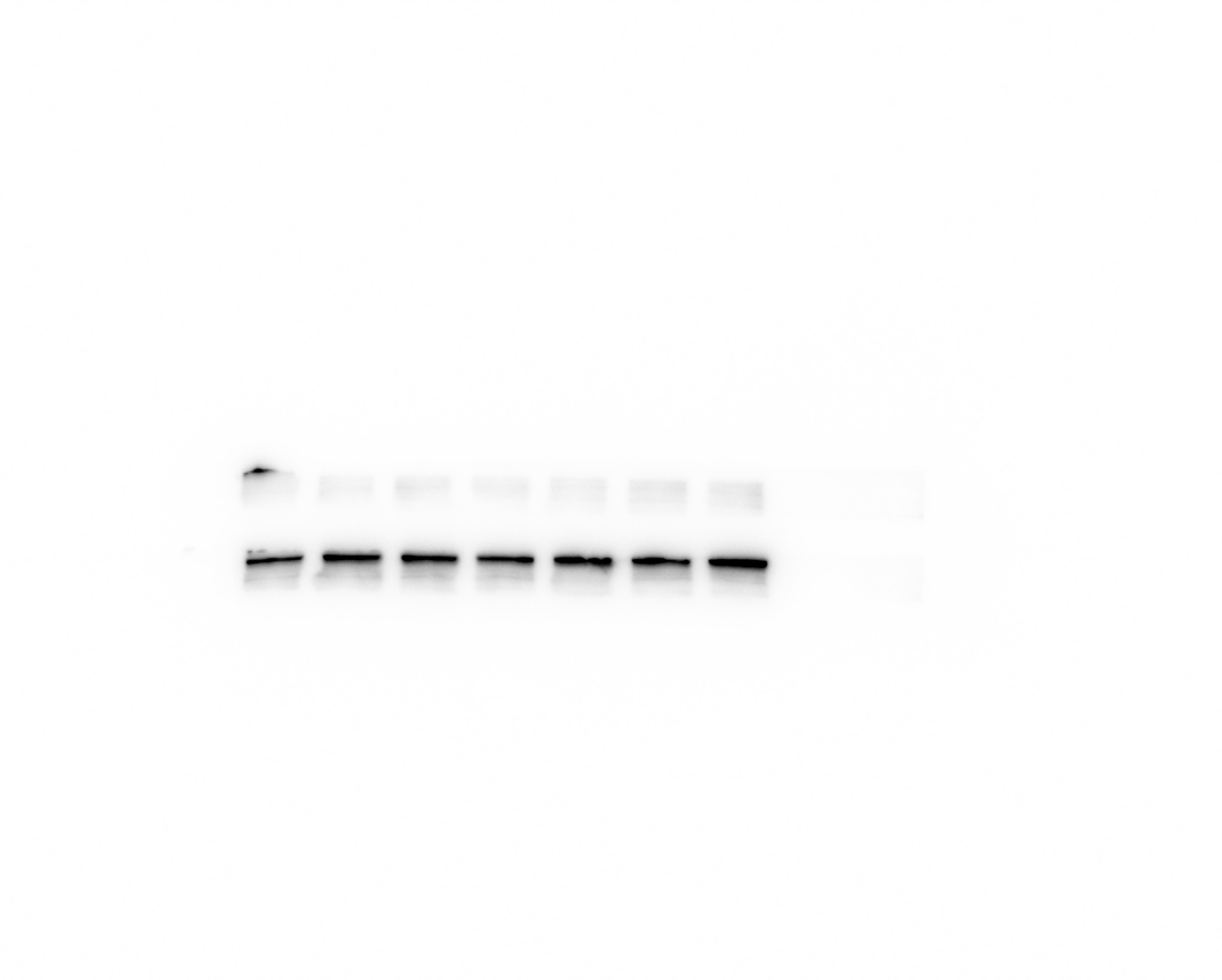


AKT (left-to-right):

Marker,

control,

H/R,

Kae + H/R,

Kae + H/R + NC,

Kae + H/R + miR-21 I,

miR-21 I,


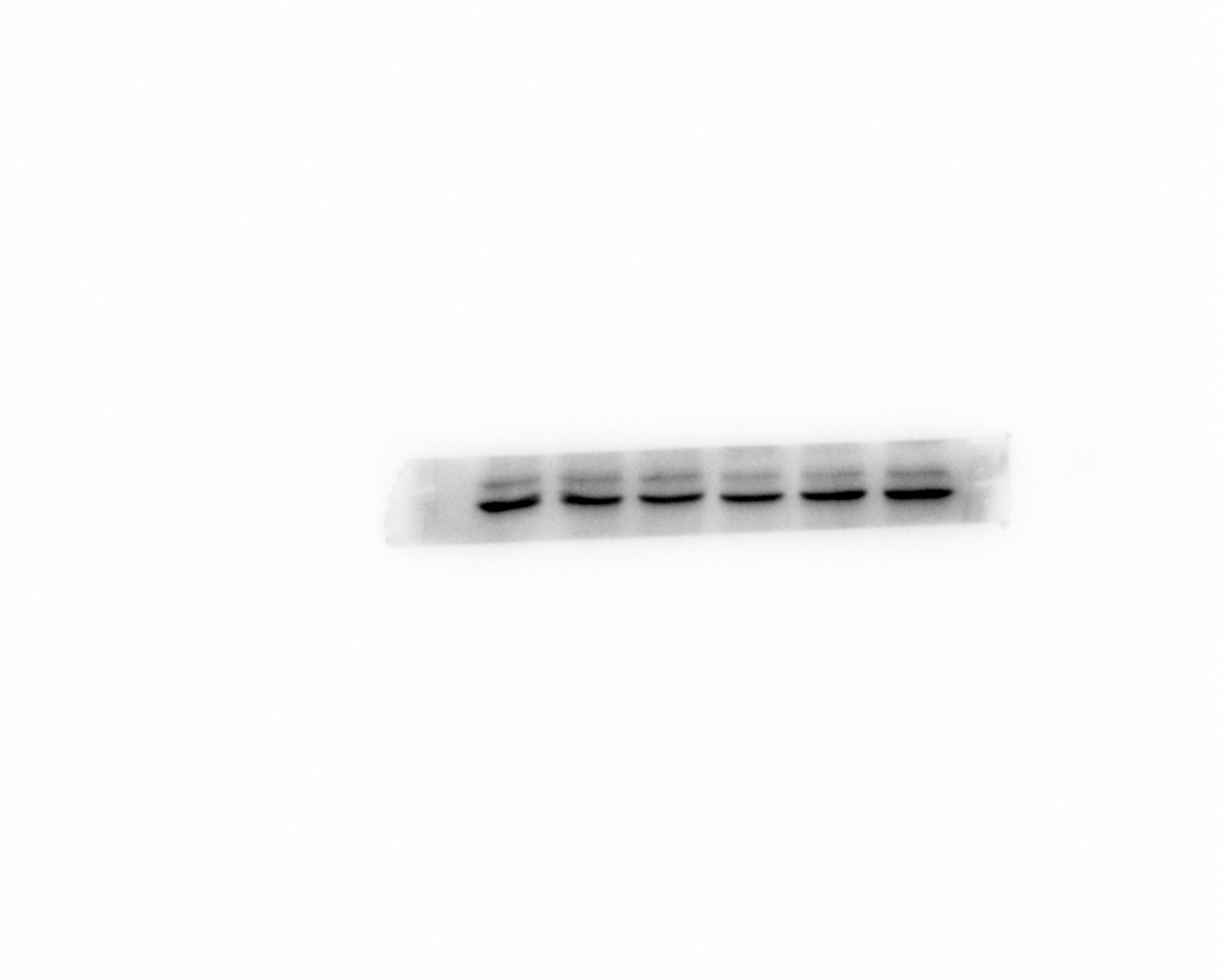
AKT (left-to-right):

control,

H/R,

Kae + H/R,

Kae + H/R + NC,

Kae + H/R + miR-21 I,

miR-21 I,


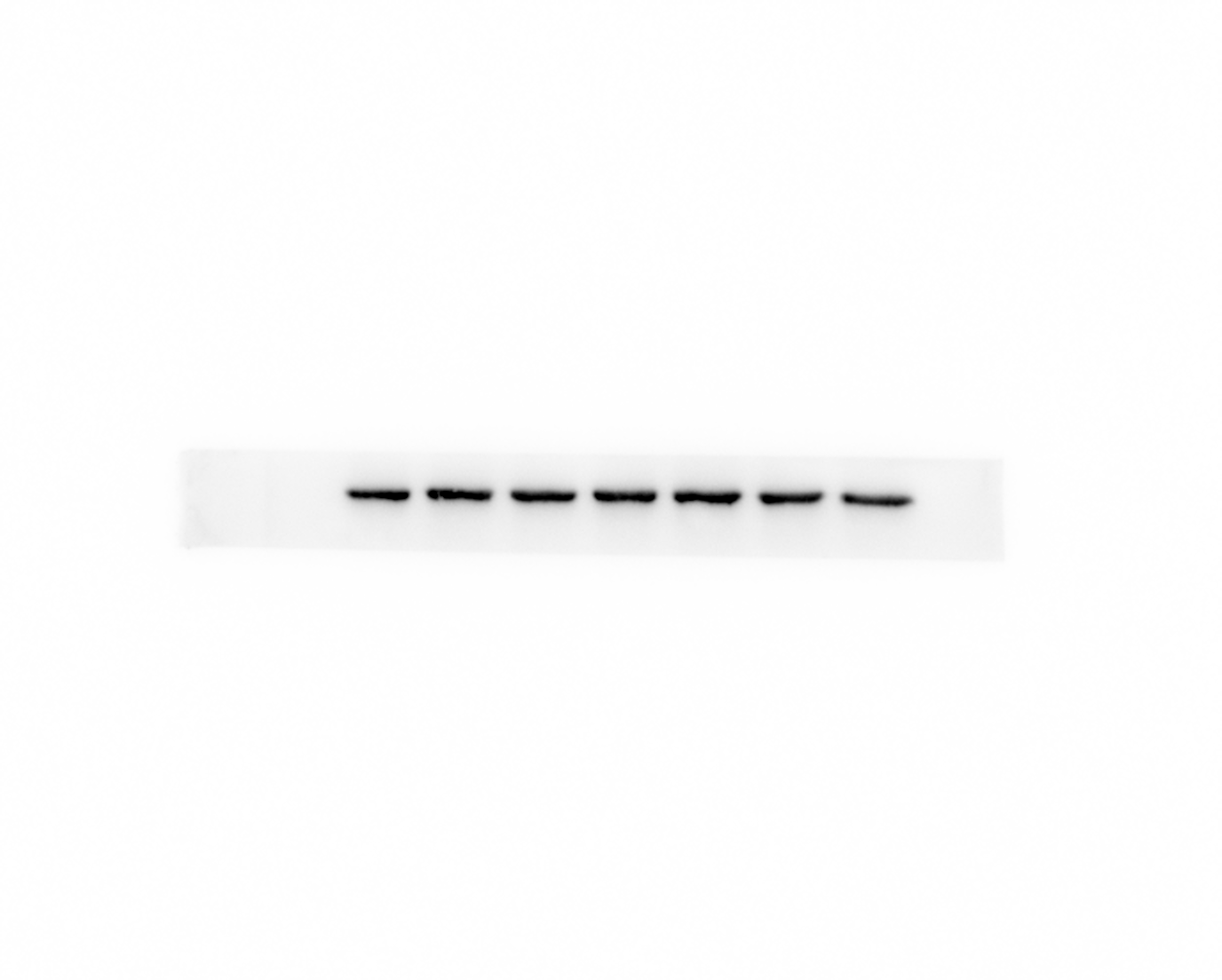


GAPDH (left-to-right):

control,

H/R,

Kae + H/R,

Kae + H/R + NC,

Kae + H/R + miR-21 I,

miR-21 I,


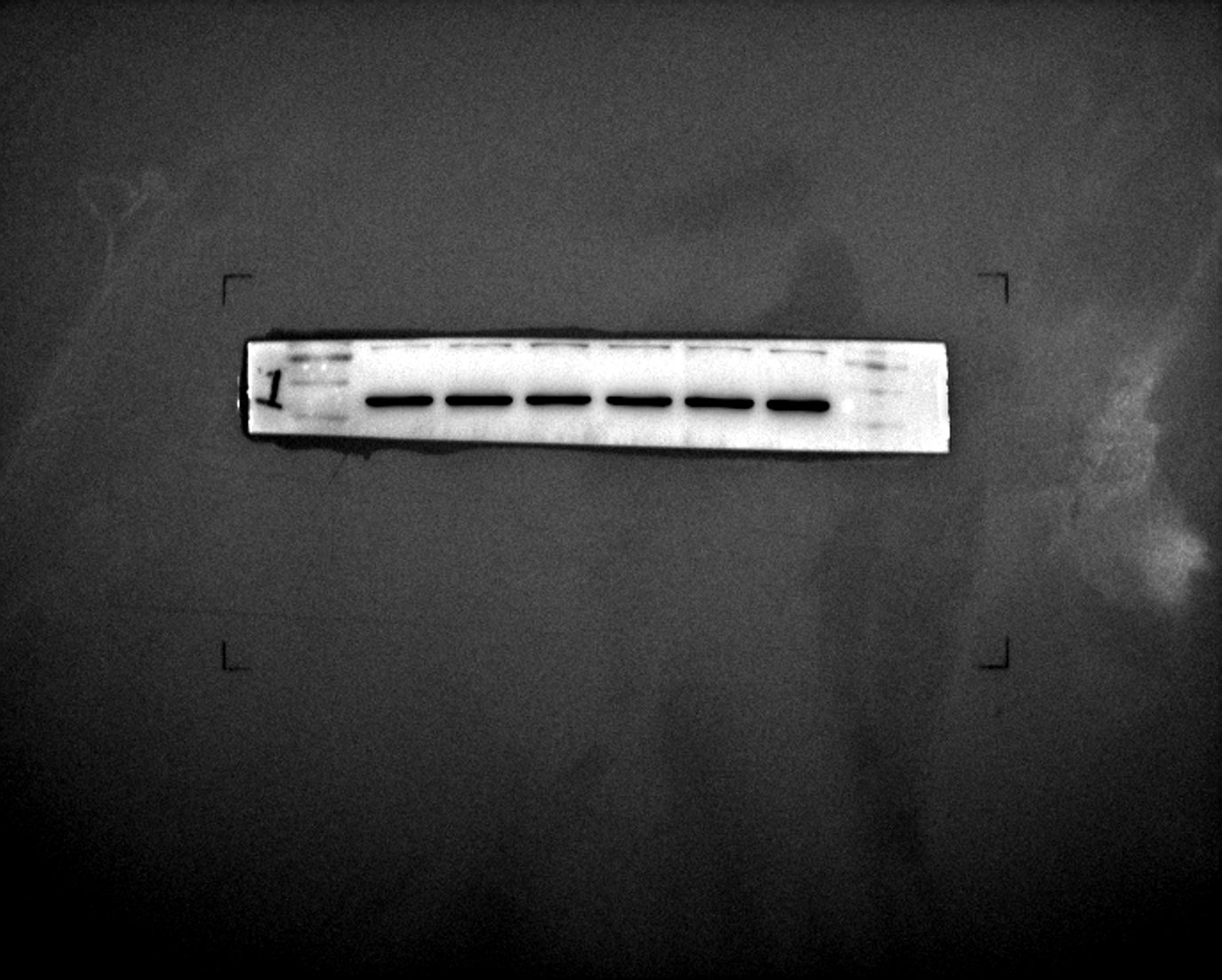


GAPDH (left-to-right):

control,

H/R,

Kae + H/R,

Kae + H/R + NC,

Kae + H/R + miR-21 I,

miR-21 I,


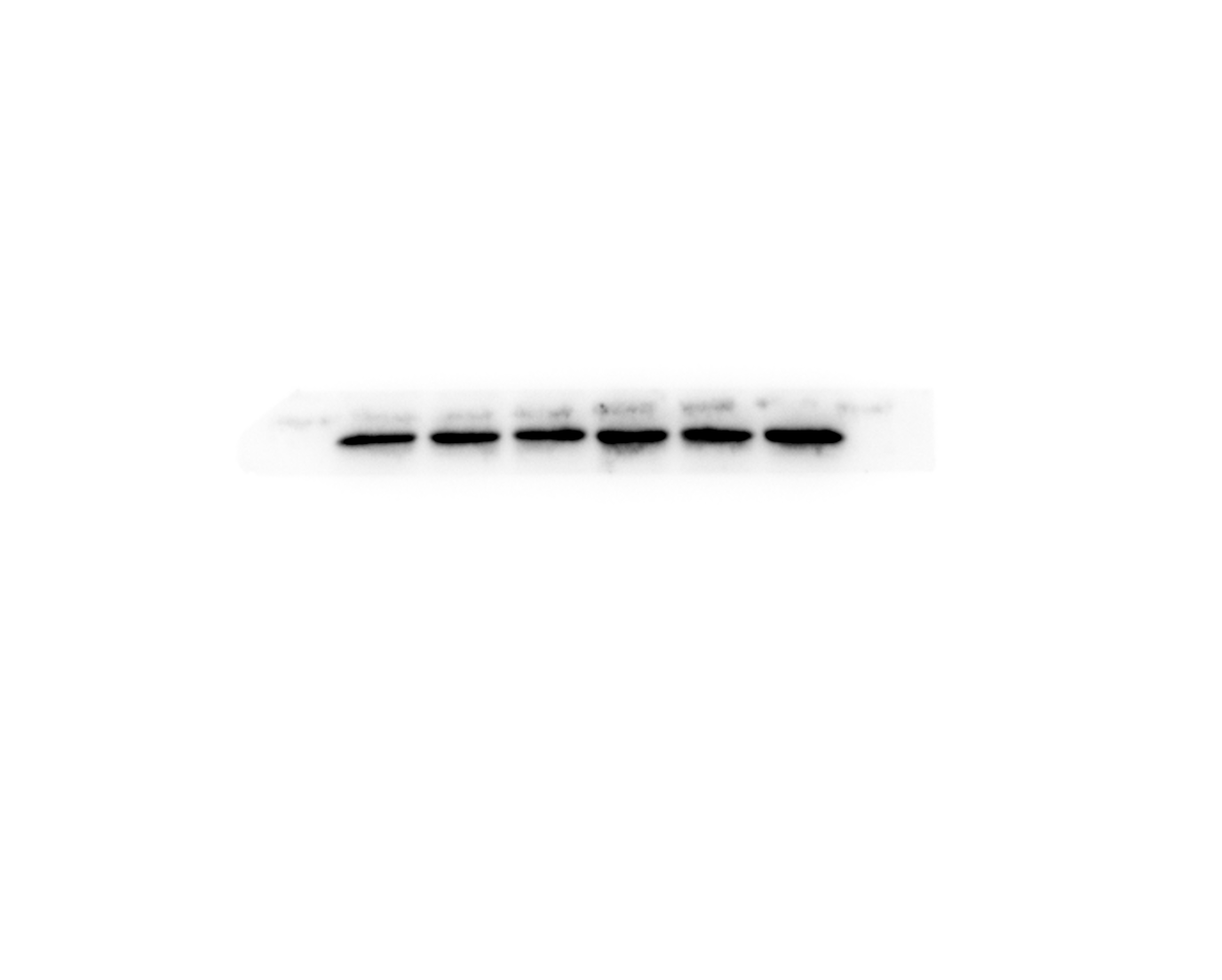


GAPDH (left-to-right):

control,

H/R,

Kae + H/R,

Kae + H/R + NC,

Kae + H/R + miR-21 I,

miR-21 I,
